# Investigating the Spectrum of Biological Activity of Substituted Quinoline-2-Carboxamides and Their Isosteres ^†^

**DOI:** 10.3390/molecules17010613

**Published:** 2012-01-10

**Authors:** Tomas Gonec, Pavel Bobal, Josef Sujan, Matus Pesko, Jiahui Guo, Katarina Kralova, Lenka Pavlacka, Libor Vesely, Eva Kreckova, Jiri Kos, Aidan Coffey, Peter Kollar, Ales Imramovsky, Lukas Placek, Josef Jampilek

**Affiliations:** 1 Department of Chemical Drugs, Faculty of Pharmacy, University of Veterinary and Pharmaceutical Sciences, Palackeho 1/3, 61242 Brno, Czech Republic; 2 Department of Ecosozology and Physiotactics, Faculty of Natural Sciences, Comenius University, Mlynska dolina Ch-2, 842 15 Bratislava, Slovakia; 3 Department of Biological Sciences, Cork Institute of Technology, Bishopstown, Cork, Ireland; 4 Institute of Chemistry, Faculty of Natural Sciences, Comenius University, Mlynska dolina Ch-2, 842 15 Bratislava, Slovakia; 5 Department of Human Pharmacology and Toxicology, Faculty of Pharmacy, University of Veterinary and Pharmaceutical Sciences, Palackeho 1/3, 61242 Brno, Czech Republic; 6 Institute of Organic Chemistry and Technology, Faculty of Chemical Technology, University of Pardubice, Studentska 573, 532 10 Pardubice, Czech Republic; 7 Pragolab s.r.o., Nad Krocinkou 55/285, 19000 Prague 9, Czech Republic

**Keywords:** quinolines, naphthalene, lipophilicity, photosynthetic electron transport inhibition, spinach chloroplasts, *in vitro* antimycobacterial activity, *in vitro* cytotoxicity

## Abstract

In this study, a series of thirty-five substituted quinoline-2-carboxamides and thirty-three substituted naphthalene-2-carboxamides were prepared and characterized. They were tested for their activity related to the inhibition of photosynthetic electron transport (PET) in spinach (*Spinacia oleracea* L.) chloroplasts. Primary *in vitro* screening of the synthesized compounds was also performed against four mycobacterial species. *N*-Cycloheptylquinoline-2-carboxamide, *N*-cyclohexylquinoline-2-carboxamide and *N*-(2-phenylethyl)quinoline-2-carboxamide showed higher activity against *M. tuberculosis* than the standards isoniazid or pyrazinamide and 2-(pyrrolidin-1-ylcarbonyl)quinoline and 1-(2-naphthoyl)pyrrolidine expressed higher activity against *M. kansasii* and *M. avium paratuberculosis* than the standards isoniazid or pyrazinamide. The most effective antimycobacterial compounds demonstrated insignificant toxicity against the human monocytic leukemia THP-1 cell line. The PET-inhibiting activity expressed by IC_50_ value of the most active compound *N-*benzyl-2-naphthamide was 7.5 μmol/L. For all compounds, the structure-activity relationships are discussed.

## 1. Introduction

The presence of an amide or thioamide (-NHCO- or -NHCS-) group is characteristic of a number of biologically active compounds, e.g., [1,2,3,4,5,6,7,8,9]. The wide spectrum of biological effects of substituted quinoline or naphthalene scaffolds includes especially antimicrobial, antineoplastics and antiviral activity [1,10,11,12,13,14,15,16,17,18,19,20,21,22,23,24,25]. In addition, some quinoline derivatives also showed noteworthy herbicidal activity [13,15,16,17,18,26] and they were also found to be uncouplers of photosynthetic phosphorylation [27]. Over 50% of commercially available herbicides act by reversibly binding to photosystem II (PS II), a membrane-protein complex in the thylakoid membranes, which catalyses the oxidation of water and the reduction of plastoquinone [28], and thereby inhibit photosynthesis [29,30,31].

Both pharmaceuticals and pesticides are designed to target particular biological functions, and in some cases these functions overlap in their molecular target sites, or they target similar processes or molecules. Modern herbicides express low toxicity against mammals and one of the reasons is that mammals lack many of the target sites for herbicide action. At present, approximately 20 mechanisms of action of herbicides are known. It was determined that inhibitors of protoporphyrinogen oxidase, 4-hydroxyphenylpyruvate dioxygenase and glutamine synthetase inhibit these enzymes both in plants and mammals. However, the consequences of inhibition of the overlapping target site can be completely different for plants and animals. Therefore a compound that has lethal action on plants may be beneficial for mammals [32]. Such chemical compounds are characterized by low toxicity on mammals as a result of quick metabolism and/or elimination of herbicide from the mammal system. Taking into consideration that mammals may also have molecular sites of action of herbicides, most pharmaceutical companies until recently had pesticide divisions, sometimes with a different name. All compounds generated by either division of the company were evaluated for both pesticide and pharmaceutical uses. In the past, some leading pesticides have become pharmaceuticals and *vice versa*. However, little information of this type was published and must usually be deduced from patent literature. One of the exceptions is fluconazole, a fungicide product discovered by the pharmaceutical sector that is now used both as a pharmaceutical and patented as a crop production chemical [32,33,34].

In the context of the previously-described azanaphtalenes [13,15,16,17,18,26] or various amides [3,4,5,6,7,8,9], new simple modifications of quinoline and naphtalene as quinoline isosteres that can trigger interesting biological activity were investigated. The compounds were tested for their photosynthesis-inhibiting activity—The inhibition of photosynthetic electron transport in spinach chloroplasts (*Spinacia oleracea* L.). The compounds were also assessed for activity against various mycobacterial species. Relationships between the structure and their *in vitro* antimycobacterial activities or/and activity related to inhibition of photosynthetic electron transport (PET) in spinach chloroplasts are discussed.

## 2. Results and Discussion

### 2.1. Chemistry

**Scheme 1:**
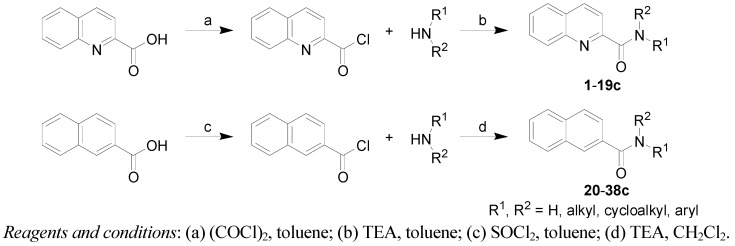
Synthesis of quinoline-2-carboxanilides **1**–**19c** and naphthalene-2-carboxamides **20**–**38c**.

All the studied compounds were prepared according to Scheme 1. Condensation of the chlorides of 2-quinaldic and 2-naphthoic acids with commercially available substituted amines yielded a series of thirty-five substituted quinoline-2-carboxamides **1**–**19c** and thirty-three substituted naphthalene-2-carboxamides **20**–**38c**. Quinoline-2-carbonyl chloride was prepared using oxalyl chloride to ensure mild conditions and prevent quinoline nucleus chloration, whereas 2-naphtoyl chloride was obtained by the classical procedure using thionyl chloride.

Hydrophobicities (log *P*) of all compounds **1**–**38c** were calculated using the commercially available program ACD/LogP (ACD/LogP ver. 1.0, Advanced Chemistry Development Inc., Toronto, Canada). The results are shown in Table 1 and Table 2. Compounds show a wide range of lipophilicities, with log *P* (ACD/LogP) values from 1.15 (compound **3**, pyrrolidinyl) to 6.98 (compound **2**, octyl) within the series of quinolinecarboxamides and from 2.10 (compound **22**, pyrrolidinyl) to 7.94 (compound **21**, octyl) within the series of naphthalenecarboxamides. Individual substituents in the amide part of the discussed compounds also result in a wide range (from −0.39 to 1.26) of electronic properties expressed as σ parameters [35,36,37,38].

**Table 1:** The calculated lipophilicities (log *P*/Clog *P*), electronic σ parameters and IC_50_ [μmol/L] values related to PET inhibition in spinach chloroplasts of quinoline-2-carboxamides **1**–**19c** in comparison with 3-(3,4-dichlorophenyl)-1,1-dimethylurea (DCMU) standard. NF = not found in literature, ND = not determined due to precipitation during the experiment or interaction with 2,6-dichlorophenol-indophenol (DCIPP).

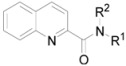					
Comp.	R^1^	R^2^	PET inhibition IC_50_ [μmol/L]	log *P* ACD/Log P	σ [35]
**1**	*i*-Pr	H	ND	2.02 ± 0.34	−0.19 [36]
**2**	–C_12_H_25_	H	573	6.98 ± 0.32	NF
**3**	–(CH_2_)_4_–		1598	1.15 ± 0.26	NF
**4**	–(CH_2_)_5_–		ND	1.72 ± 0.26	NF
**5**	*c*-Pn	H	762	2.63 ± 0.33	−0.20 [36]
**6**	*c*-Hx	H	1041	3.20 ± 0.33	−0.15 [36]
**7**	*c*-Hp	H	ND	3.76 ± 0.33	NF
**8**	*c*-Oc	H	415	4.33 ± 0.33	NF
**9**	Ph	H	85.1	2.90 ± 0.34	0.60 [36]/0
**10**	Bn	H	59.4	2.91 ± 0.35	0.22 [36]
**11**	–C_2_H_4_Ph	H	ND	3.33 ± 0.33	0.08 [36]
**12a**	2-OH-Ph	H	16.3	2.54 ± 0.36	−0.09
**12b**	3-OH-Ph	H	ND	2.55 ± 0.36	0.12
**12c**	4-OH-Ph	H	ND	2.15 ± 0.35	−0.37
**13a**	2-OCH_3_-Ph	H	ND	2.80 ± 0.36	−0.39 [37]
**13b**	3-OCH_3_-Ph	H	ND	3.06 ± 0.36	0.12
**13c**	4-OCH_3_-Ph	H	ND	2.85 ± 0.36	−0.27
**14a**	2-CH_3_-Ph	H	142.9	3.36 ± 0.34	NF
**14b**	3-CH_3_-Ph	H	100.6	3.36 ± 0.34	−0.07
**14c**	4-CH_3_-Ph	H	ND	3.36 ± 0.34	−0.17
**15a**	2-F-Ph	H	98.1	2.86 ± 0.44	0.24 [38]
**15b**	3-F-Ph	H	86.9	3.38 ± 0.44	0.34
**15c**	4-F-Ph	H	75.3	3.34 ± 0.44	0.06
**16a**	2-Cl-Ph	H	56.3	3.41 ± 0.36	0.20 [38]
**16b**	3-Cl-Ph	H	91.9	3.93 ± 0.37	0.37
**16c**	4-Cl-Ph	H	147.6	3.89 ± 0.36	0.23
**17a**	2-Br-Ph	H	92.2	3.58 ± 0.44	0.21 [38]
**17b**	3-Br-Ph	H	409.0	4.10 ± 0.44	0.39
**17c**	4-Br-Ph	H	307.9	4.06 ± 0.44	0.23
**18a**	2-CF_3_-Ph	H	109.4	4.09 ± 0.42	NF
**18b**	3-CF_3_-Ph	H	329.5	4.25 ± 0.42	0.43
**18c**	4-CF_3_-Ph	H	ND	3.91 ± 0.41	0.74
**19a**	2-NO_2_-Ph	H	ND	3.15 ± 0.38	0.80 [38]
**19b**	3-NO_2_-Ph	H	ND	3.28 ± 0.38	0.71
**19c**	4-NO_2_-Ph	H	ND	3.36 ± 0.38	1.26
**DCMU**	–	–	1.9	–	–

**Table 2:** The calculated lipophilicities (log *P*/Clog *P*), electronic σ parameters and IC_50_ [μmol/L] values related to PET inhibition in spinach chloroplasts of naphthalene-2-carboxamides **20**–**38c** in comparison with 3-(3,4-dichlorophenyl)-1,1-dimethylurea (DCMU) standard. NF = not found in literature, ND = not determined due to precipitation during the experiment or interaction with 2,6-dichlorophenol-indophenol (DCIPP).

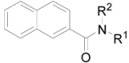					
Comp.	R^1^	R^2^	PET inhibition IC_50_ [μmol/L]	log *P* ACD/Log P	σ [35]
**20**	*i*-Pr	H	353	2.97 ± 0.28	−0.19 [36]
**21**	–C_12_H_25_	H	845	7.94 ± 0.28	NF
**22**	–(CH_2_)_4_–		ND	2.10 ± 0.23	NF
**23**	–(CH_2_)_5_–		ND	2.67 ± 0.23	NF
**24**	*c*-Pn	H	ND	3.59 ± 0.28	−0.20 [36]
**25**	*c*-Hx	H	ND	4.15 ± 0.28	−0.15 [36]
**26**	*c*-Hp	H	216	4.21 ± 0.28	NF
**27**	*c*-Oc	H	688	5.28 ± 0.28	NF
**28**	Ph	H	20.7	3.85 ± 0.29	0.60 [36]/0
**29**	Bn	H	7.5	3.87 ± 0.28	0.22 [36]
**30**	–C_2_H_4_Ph	H	ND	4.28 ± 0.29	0.08 [36]
**31b**	3-OH-Ph	H	ND	3.50 ± 0.31	0.12
**31c**	4-OH-Ph	H	ND	3.11 ± 0.30	−0.37
**32a**	2-OCH_3_-Ph	H	763.0	3.75 ± 0.32	−0.39 [37]
**32b**	3-OCH_3_-Ph	H	306.2	4.01 ± 0.32	0.12
**32c**	4-OCH_3_-Ph	H	ND	3.80 ± 0.32	−0.27
**33a**	2-CH_3_-Ph	H	ND	4.31 ± 0.30	NF
**33b**	3-CH_3_-Ph	H	ND	4.31 ± 0.30	−0.07
**33c**	4-CH_3_-Ph	H	ND	4.31 ± 0.30	−0.17
**34a**	2-F-Ph	H	ND	3.82 ± 0.40	0.24 [38]
**34b**	3-F-Ph	H	ND	4.34 ± 0.41	0.34
**34c**	4-F-Ph	H	ND	4.30 ± 0.41	0.06
**35a**	2-Cl-Ph	H	ND	4.36 ± 0.32	0.20 [38]
**35b**	3-Cl-Ph	H	81.0	4.88 ± 0.33	0.37
**35c**	4-Cl-Ph	H	ND	4.84 ± 0.32	0.23
**36a**	2-Br-Ph	H	ND	4.54 ± 0.40	0.21 [38]
**36b**	3-Br-Ph	H	102.8	5.06 ± 0.41	0.39
**36c**	4-Br-Ph	H	ND	5.02 ± 0.41	0.23
**37b**	3-CF_3_-Ph	H	309.4	5.20 ± 0.39	0.43
**37c**	4-CF_3_-Ph	H	ND	4.86 ± 0.37	0.74
**38a**	2-NO_2_-Ph	H	ND	4.10 ± 0.34	0.80 [38]
**38b**	3-NO_2_-Ph	H	633.3	4.24 ± 0.34	0.71
**38c**	4-NO_2_-Ph	H	260.0	4.31 ± 0.34	1.26
**DCMU**	–	–	1.9	–	–

### 2.3. Biological Activities

The compounds under investigation could be divided into two groups based on their chemical structure: Group 1 included quinoline-2-carboxamides **1**–**19c**; Group 2 contained naphthalene-2-carboxamides **20**–**38c**. Compounds within both series can be also divided according to whether they contain an aromatic or a non-aromatic amine. The compounds showed a wide range of biological activities and some interesting structure-activity relationships were observed. All the results are summarized in Table 1, Table 2, Table 3. Generally, all the discussed compounds exhibited problematic solubility, but quinoline derivatives generally possess better aqueous solubility in comparison to the naphthamides.

**Table 3:** *In vitro* antimycobacterial activity (IC_90_) of compounds **1**–**3**, **5**–**7**, **11**, **14b**, **22** and **32a** in comparison with standards isoniazid (INH) and pyrazinamide (PZA), *in vitro* cytotoxicity assay (LD_50_) of compounds **3**, **7**, **11** and **22** and calculated selectivity index (SI). ND = not determined; used IC_90_ for calculation of SI is marked by *.

Comp.	MIC/IC_90_ [µmol/L]					LD_50_ [µmol/L]	SI [LD_50_/IC_90_]
	*M. tuberculosis ^#^*	*M. avium* complex		*M*.*avium paratuberculosis*	*M. kansasii*		
**1**	280		583	1167	1167	ND	–
**2**	367		367	734	734	ND	–
**3**	552		552	111 *	111 *	>100	>0.90
**5**	491		491	491	491	ND	–
**6**	125		520	520	520	ND	–
**7**	111 *		466	223	466	62 ± 4.5	0.56
**11**	109 *		452	905	452	>100	>0.92
**14b**	469		469	939	469	ND	–
**22**	554		554	111 *	111 *	>100	>0.90
**32a**	901		451	216	451	ND	–
**INH**	>729		<72.9	>729	<72.9	ND	–
**PZA**	>812		>812	>812	>812	ND	–

*^#^* Clinical isolate of *M. tuberculosis* CUH071 (Cork University Hospital TB lab), with partial INH and PZA resistance.

#### 2.3.1. Inhibition of Photosynthetic Electron Transport (PET) in Spinach Chloroplasts

**Figure 1:**
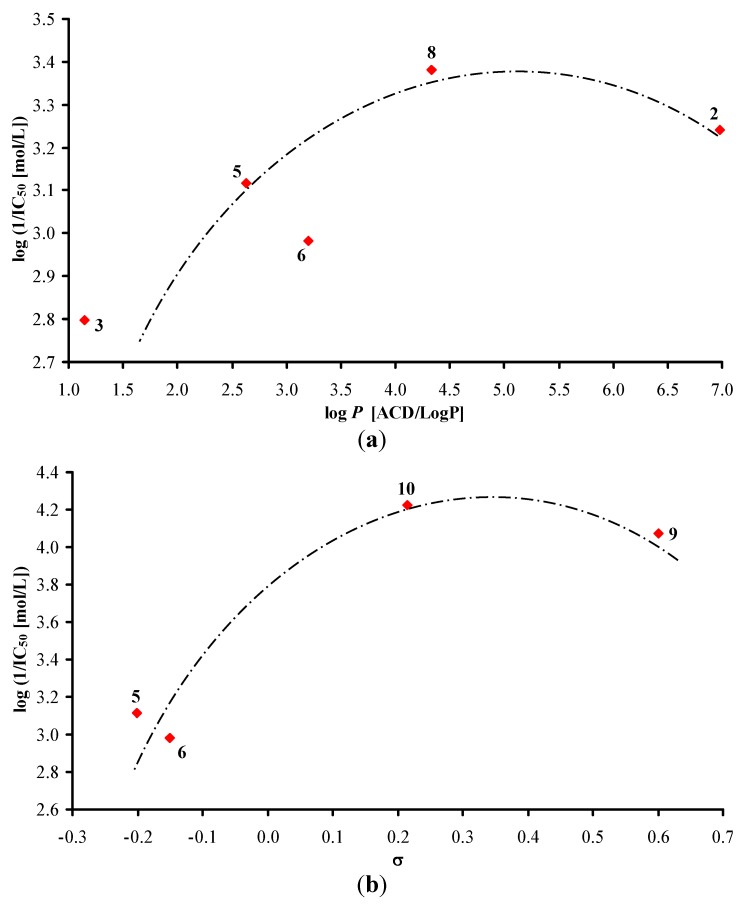
Relationships between PET inhibition log (1/IC_50_) [mol/L] in spinach chloroplasts and lipophilicity (**a**) or *N*-substituent electronic σ parameters (**b**) of studied compounds **1**–**10**.

**Figure 2:**
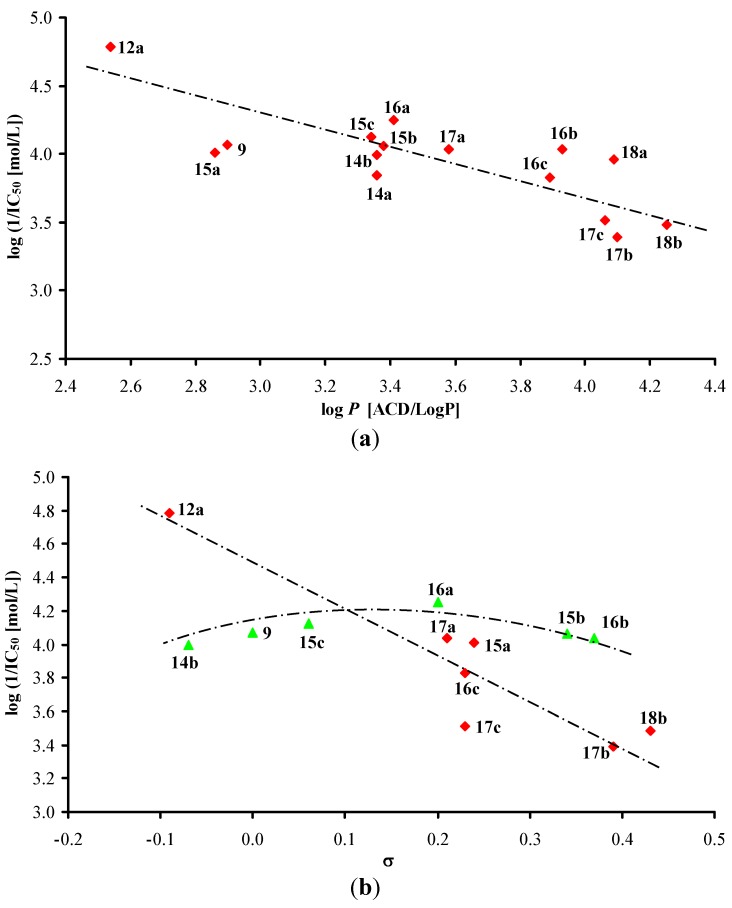
Relationships between PET inhibition log (1/IC_50_) [mol/L] in spinach chloroplasts and lipophilicity (**a**) or *N*-substituent electronic Hammett’s σ parameters (**b**) of studied compounds **9**, **12a**–**19c**.

**Figure 3:**
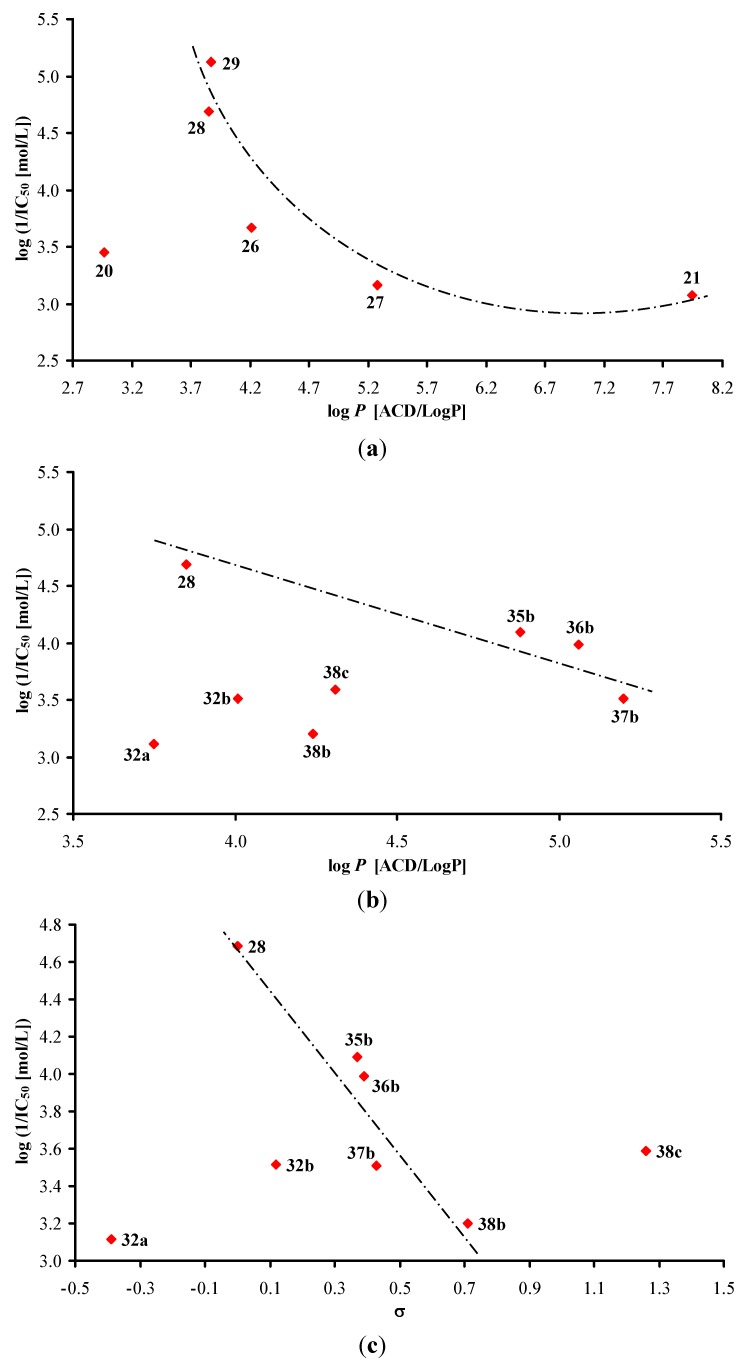
Relationships between PET-inhibition log (1/IC_50_) [mol/L] in spinach chloroplasts and lipophilicity (**a**, **b**) or *N*-substituent electronic σ parameters (**c**) of studied naphthalene-2-carboxamides **20**–**38c**.

The activity of all the evaluated derivatives related to inhibition of photosynthetic electron transport (PET) in spinach (*Spinacia oleracea* L.) chloroplasts was moderate or rather low relative to the standard, see Table 1 and Table 2. *N-*(2-Hydroxyphenyl)quinoline-2-carboxamide (**12a**) expressed the highest PET-inhibiting activity (IC_50_ = 16.3 µmol/L) within the series of quinolinecarboxamides whereas *N-*benzyl-2-naphthamide (**29**) from the second set investigated was two-fold more effective with a PET inhibition IC_50_ value of 7.5 µmol/L. The PET-inhibiting activity was expressed by the negative logarithm of the IC_50_ value (compound concentration in mol/L causing 50% inhibition of PET). Despite the relatively low inhibitory activity of the rest studied compounds, correlations between log (1/IC_50_) and the lipophilicity expressed as log *P* (ACD/LogP) or electronic properties expressed as Hammett’s σ parameters of the individual substituents in compounds **1**–**38c** were performed, see Figure 1, Figure 2 and Figure 3. Generally, non-aromatic *N*-substituents showed lower PET-inhibiting activity than anilides.

The PET-inhibiting activity of the evaluated quinoline derivatives (Group 1) is summarized in Table 1. According to both Figure 1, where all compounds except substituted phenyl rings are illustrated, it can be stated that the dependence of PET-inhibiting activity on the lipophilicity showed a parabolic course. Cyclic non-aromatic *N*-substituents are illustrated in Figure 1a, where the most active compound *N*-cyclooctyl **8** has a lipophilicity optimum at log *P* = 4.33. The dependence of PET-inhibiting activity on electronic constants σ of non-aromatic as well as phenyl **9** and benzyl **10** substituents obtained from literature [36] seems to also follow a parabolic course, see Figure 1b. Benzyl derivative **10** showed an optimum of weak electron-withdrawing effect influencing the electronic density of the amido moiety. It is evident that bulkiness of the *N*-substituents did not influence PET-inhibiting activity.

Figure 2 shows the correlations between log (1/IC_50_) and the lipophilicity or Hammett’s σ parameters of the individual anilide substituents in compounds **9**, **12a**–**19c**. Based on the results obtained it is not possible to decide whether some of the *ortho*-, *meta*- or *para*-positions are preferred from the PET-inhibiting activity point of view. Nevertheless, according to Figure 2a it can be stated that the PET inhibition showed linear decrease with increasing lipophilicity and the lipophilicity of the compounds was decisive for PET inhibition:


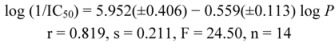
(1)

On the other hand, the biological activity was also affected by electronic σ properties of these anilide substituents, see Figure 2b.

In general, the dependence of log (1/IC_50_) on σ reflects two trends. The first, *i.e.*, compounds with extreme electron σ effects, show a similar trend as in case of lipophilicity and thus, the activity decreases with increasing σ value. The second trend is a parabolic course with an optimum activity for compound **16a** (2-Cl-Ph; σ = 0.20). In this case the σ values of the corresponding compounds ranged from −0.07 to 0.37, but the differences between activities of the compounds expressed as IC_50_ values were relatively low and they ranged from 100.6 (**14b**) to 56.3 μmol/L (**16a**). However, the importance of the lipophilicity of the anilide substituent was unambiguously much more significant for the inhibitory activity (IC_50_ [mol/L]) of the studied compounds than the electronic properties of the substituent.

The most active compound from the series Group 1 was **12a** (R = 2-OH, IC_50_ = 16.3 μmol/L). The result indicates that PET inhibition could be associated with additional interaction of the phenol moiety with photosynthetic proteins. This compound can be understood as a bioisoster of 2-[(2-hydroxyphenylimino)methyl]quinolin-8-ol that expresses high herbicide effect [18].

Generally, the activity of the evaluated naphthalene derivatives (Group 2) related to PET inhibition in spinach (*Spinacia oleracea* L.) chloroplasts seems to be higher than that of the corresponding quinoline isosters (Table 2). The PET inhibition of 34 of the 68 compounds could not be determined due to their precipitation during the experiments. With respect to these small but closed specifically substituted groups of compounds some structure-activity relationships (SAR) can be proposed.

Figure 3a illustrates the exponential decay of PET-inhibiting activity on the lipophilicity increase of all naphthalene derivatives (compounds **20**, **21**, **26**–**29**) except substituted phenyl moieties. Dependence of the activity on the electronic properties could not be performed due to small amount of data. As mentioned above, PET-inhibiting activity is not influenced by bulkiness of the *N*-substituents. Benzyl derivative **29** showed higher activity (IC_50_ = 7.5 μmol/L) than unsubstituted phenyl derivative **28** (IC_50_ = 20.7 μmol/L), similarly to quinolinecarboxamides [IC_50_ = 59.4 μmol/L (**10**) and 85.1 μmol/L (**9**), respectively].

Dependence of PET inhibition on the lipophilicity of all compounds with the aromatic *N*-substituents is shown on Figure 3b. It is evident that unsubstituted phenyl **28** derivative expressed the highest PET inhibition; and this decreases as lipophilicity increases. This is observed for lipophilic *meta*-substituted derivatives **35b** (3-Cl), **36b** (3-Br) and **37b** (3-CF_3_), contrary to the quinaldinanilides, and it is not valid for methoxy and nitro moieties (**32a**/**b**, **38b**/**c**). This lower inhibitory activity of compounds **32a** (2-OCH_3_-Ph), **32b** (2-OCH_3_-Ph) and **38c** (4-NO_2_-Ph) could be a result of the limited solubility of the compounds, which is typical for -OCH_3_ and -NO_2_ substituents. Figure 3c shows PET inhibition of naphtanilides on Hammett’s σ parameters of the individual substituents. Based on the results from Figure 3c it can be concluded that PET-inhibiting activity is strongly decreased by the electron-withdrawing effect of substituents in the anilide part of the molecule: **29** (Ph) >> **35b** (3-Cl) > **36b** (3-Br) > **37b** (3-CF_3_) > **38b** (3-NO_3_), especially in *meta*-position, contrary to the SAR of quinaldinanilides discussed above.

#### 2.3.3. *In Vitro* Antimycobacterial Evaluation

Although all the compounds were evaluated for their *in vitro* antimycobacterial activity against *M. tuberculosis* (clinical isolate with partial INH and PZA resistance) and other atypical mycobacterial strains, most compounds did not show any activity due to their low solubility and precipitation during the experiments. Only the 10 compounds presented in Table 3 showed actimycobacterial activities. *N*-Cycloheptylquinoline-2-carboxamide (**7**) and *N*-(2-phenylethyl)quinoline-2-carboxamide (**11**) showed high activity against *M. tuberculosis*, whereas 2-(pyrrolidin-1-ylcarbonyl)quinoline (**3**) and 1-(2-naphthoyl)pyrrolidine (**22**) expressed high activity against *M. avium paratuberculosis* and *M. kansasii*. Compounds **6**, **1** and **32a** also showed noteworthy activity. Nevertheless, no thorough structure-activity relationships could be established.

**Figure 4:**
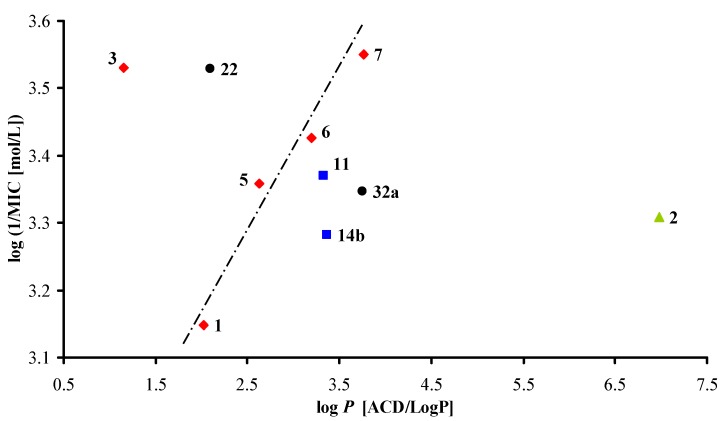
Dependence of *in vitro* average antimycobacterial activity against *Mycobacterium* sp. (log 1/MIC [mol/L]) on lipophilicity expressed as log *P* of the studied quinaldinamides. (red rhomb = branched alkyl quinaldinamides, green triangle = unbranched long-chain alkyl quinaldinamide, blue square = aromatic quinaldinamides, black point = naphtamides).

According to the results, it can be generally concluded that quinaldinamides (Group 1) possessed higher activity than corresponding naphtamides (Group 2), and anilides seem to be less effective than amides, e.g., highly effective compound **11** and non-active phenyl derivative **9 **(not-discussed). Figure 4 shows dependence of the average of antituberculosis/antimycobacterial activities expressed as log 1/MIC [mol/L] on lipophilicity expressed as log *P*. Based on these results, it can be concluded that activity increases as lipophilicity increases. Also, it seems that the increase in antituberculosis activity is connected with the increase in the bulkiness of individual *N*-substituents within the series of cycloalkane, (*i.e.*, cycloheptyl **7** > cyclohexyl **6** > cyclopentyl **5**). As cyclooctyl derivative **8** demonstrated no activity, it can be concluded that cycloheptyl compound **7** showed the maximum antituberculosis efficacy within this type of compound and under these testing conditions. This decrease might have also actually been caused by decreased solubility of compound **8** in the test media (precipitation occurred). It can be speculated that substituents which are bulkier than cyclooctyl could further potentiate the antimycobacterial activity. But the testing conditions for these more lipophilic compounds would have to be changed to prevent the precipitation during the dilution of samples.

Branching within the *N*-substituents seems also very important, especially from the point of view of antitubercular *versus* antimycobacterial activity. Unbranched long-chain alkyl dodecyl compound **2** or ethylphenyl one **11** as well as isopropyl compound **1** and other cycloalkyl moietis branched in the α position of individual *N*-substituents, seem to be the most advantageous for antituberculosis activity, while *N*,*N*-disubstitution, e.g., compounds **3** and **22**, is fundamental for high efficacy against the atypical mycobacterial strains *M*.*avium paratuberculosis* and *M. kansasii*.

#### 2.3.4. *In Vitro* Cytotoxicity Assay

The most effective antimycobacterial compounds **11**, **7**, **3** and **22** were tested for their *in vitro* cytotoxicity LD_50_ (µmol/L), and subsequently the Selectivity Index, *i.e.*, the ratio of cell toxicity (LD_50_) to activity (MIC), was obtained; the results are presented in Table 3. The LD_50_ exact values of compounds **3**, **11** and **22** could not be determined due to their limited solubility and their precipitation from solution during the tests in concentration higher than 100 µmol/L, but the highest dose achieved in the medium (100 µmol/L) did not lead to the 100% lethal effect on THP-1 cells. All the evaluated compounds demonstrated low toxicity against the human monocytic leukemia THP-1 cell line (e.g., LD_50_ of oxaliplatin 1.7 ± 6.4 and camptothecin 0.16 ± 0.07 assessed in this line formerly showed much lower values). It can be drawn from Table 3 that compounds **3**, **7** and **11** from Group 1 and compound **22** from Group 2 showed the highest inhibition activity and also moderate cytotoxicity against THP-1 cells; compound **7** expressed the highest toxicity within the series of compounds, but it can not be considered toxic as its LD_50_ value (62 ± 4.5) is fairly high. Based on these observations it can be concluded that the discussed amides **11**, **3** and **22** can be considered as promising agents for subsequent design of novel antitubercular/antimycobacterial agents.

## 3. Experimental

### 3.1. General

All reagents were purchased from Aldrich. Kieselgel 60, 0.040–0.063 mm (Merck, Darmstadt, Germany) was used for column chromatography. TLC experiments were performed on alumina-backed silica gel 40 F254 plates (Merck, Darmstadt, Germany). The plates were illuminated under UV (254 nm) and evaluated in iodine vapour. The melting points were determined on Kofler hot-plate apparatus HMK (Franz Kustner Nacht KG, Dresden, Germany) and are uncorrected. Infrared (IR) spectra were recorded on a Smart MIRacle™ ATR ZnSe for Nicolet™ Impact 410 FT-IR spectrometer (Thermo Scientific, USA). The spectra were obtained by accumulation of 256 scans with 2 cm^−1^ resolution in the region of 4,000–600 cm^−1^. All ^1^H and ^13^C NMR spectra were recorded on a Bruker Avance III 400 MHz FT-NMR spectrometer (400 MHz for ^1^H and 100 MHz for ^13^C, Bruker Co., Karlsruhe, Germany). Chemicals shifts are reported in ppm (d) using internal Si(CH_3_)_4_ as the reference with diffuse, easily exchangeable signals being omitted. Mass spectra were measured using a LTQ Orbitrap Hybrid Mass Spectrometer (Thermo Electron Corporation, USA) with direct injection into an APCI source (400 °C) in the positive mode.

### 3.2. Synthesis

#### 3.2.1. General Procedure for Synthesis of Carboxamide Derivatives **1**–**19c**

2-Quinaldic acid (1 g, 5.8 mmol) was suspended in dry toluene (15 mL) at room temperature and oxalyl chloride (1 mL, 1.61 g, 12.7 mmol, 2.2 eq.) was added dropwise. The reaction mixture was stirred for 30 min at the same temperature and then DMF (2 drops) was added. The mixture was stirred for 24 h and then evaporated to dryness. The residue was washed with petroleum ether and used directly in the next step. Into the solution of 2-quinaldic acid chloride in dry toluene (15 mL), triethylamine (4.5 mL, 2.92 g, 32.5 mmol) and corresponding substituted aniline (5.8 mmol) were added dropwise. The mixture was stirred at room temperature for 24 h after which the solvent was removed under reduced pressure. The residue was extracted with CHCl_3_. Combined organic layers were washed with water and saturated aqueous solution of NaHCO_3_ and dried over anhydrous MgSO_4_. The solvent was evaporated to dryness under reduced pressure. The crude product was recrystallized from isopropanol or EtOAc. The studied compounds **1**–**19c** are presented in Table 1.

*N-Isopropylquinoline-2-carboxamide* (**1**). Yield 40%; Mp. 75–76 °C; IR (Zn/Se ATR, cm^−1^): 3,339*m*, 3,052*w*, 2,925*w*, 2,852*w*, 1,676*s*, 1,589*m*, 1,556*m*, 1,525*s*, 1,501*m*, 1,434*w*, 1,418*w*, 1,205*w*, 1,119*m*, 903*w*, 841*w*, 770*m*, 747*m*, 686*w*, 666*w*; ^1^H-NMR (DMSO-*d*_6_), δ: 8.60 (d, *J* = 8.0 Hz, 1H), 8.54 (d, *J* = 8.5 Hz, 1H), 8.16 (d, *J* = 8.5 Hz, 2H), 8.05 (d, *J* = 8.3 Hz, 1H), 7.80–7.89 (m, 1H), 7.64–7.74 (m, 1H), 4.03–4.28 (m, 1H), 1.23 (d, *J* = 6.8 Hz, 6H); ^13^C-NMR (DMSO-*d*_6_), δ: 163.02, 150.32, 145.96, 137.83, 130.45, 129.18, 128.76, 128.07, 127.98, 118.65, 40.94, 22.27; HR-MS: for C_13_H_15_N_2_O [M+H]^+^ calculated 215.1179 *m/z*, found 215.1139 *m/z*.

*N-Dodecylquinoline-2-carboxamide* (**2**). Yield 24%; Mp. 45–46 °C; IR (Zn/Se ATR, cm^−1^): 3,411*s*, 3,052*w*, 2,983*w*, 2,901*w*, 1,671*s*, 1,589*w*, 1,561*m*, 1,521*m*, 1,492*m*, 1,450*w*, 1,423*s*, 1,201*w*, 1,131*w*, 1,107*w*, 1,074*w*, 1,054*w*, 907*w*, 841*m*, 768*w*, 735*m*, 696*m*; ^1^H-NMR (DMSO-*d*_6_), δ: 8.88 (t, *J* = 6.0 Hz, 1H), 8.53 (d, *J* = 8.3 Hz, 1H), 8.15 (d, *J* = 8.5 Hz, 1H), 8.11 (d, *J* = 8.5 Hz, 1H), 8.05 (d, *J* = 8.0 Hz, 1H), 7.81–7.86 (m, 1H), 7.65–7.71 (m, 1H), 3.31–3.38 (m, 2H), 1.54 (quin, *J* = 6.9 Hz, 2H), 1.10–1.31 (m, 18H), 0.78 (t, *J* = 6.9 Hz, 3H); ^13^C-NMR (DMSO-*d*_6_), δ: 163.78, 150.29, 145.99, 137.72, 130.37, 129.13, 128.76, 128.04, 127.89, 118.60, 38.96, 31.30, 29.24, 29.06, 29.03, 29.02, 29.00, 28.81, 28.73, 26.49, 22.09, 13.89; HR-MS: for C_22_H_33_N_2_O [M+H]^+^ calculated 341.2587 *m/z*, found 341.2599 *m/z*.

*2-(Pyrrolidin-1-ylcarbonyl)quinoline* (**3**) [39]. Yield 41%; Mp. 71–72 °C; IR (Zn/Se ATR, cm^−1^): 3,291*m*, 3,064*w*, 2,919*s*, 2,844*w*, 1,644*s*, 1,561*w*, 1,532*s*, 1,498*m*, 1,467*m*, 1,438*w*, 1,422*m*, 1,344*w*, 1,324*w*, 1,201*w*, 1,168*m*, 1,070*m*, 1,009*w*, 903*m*, 842*m*, 774*s*, 735*m*, 678*m*; ^1^H-NMR (DMSO-*d*_6_), δ: 8.46 (d, *J* = 8.5 Hz, 1H), 8.03 (t, *J* = 9.2 Hz, 2H), 7.77–7.83 (m, 2H), 7.66 (ddd, *J* = 8.0 Hz, *J* = 6.9 Hz, *J* = 1.1 Hz, 1H), 3.67 (t, *J* = 6.4 Hz, 2H), 3.55 (t, *J* = 6.5 Hz, 2H), 1.78–1.89 (m, 4H); ^13^C-NMR (DMSO-*d*_6_), δ: 165.56, 154.22, 145.87, 137.07, 130.14, 129.15, 127.90, 127.75, 127.64, 120.41, 48.46, 46.39, 26.05, 23.59; HR-MS: for C_14_H_15_N_2_O [M+H]^+^ calculated 227.1179 *m/z*, found 227.1203 *m/z*.

*2-(Piperidin-1-ylcarbonyl)quinoline* (**4**) [40]. Yield 59%; Mp. 83–84 °C; IR (Zn/Se ATR, cm^−1^): 3,327*s*, 3,023*w*, 2,934*w*, 1,655*s*, 1,561*w*, 1,522*m*, 1,495*m*, 1,454*m*, 1,426*s*, 1,360*m*, 1,331*w*, 1,230*w*, 1,209*w*, 1,156*m*, 1,074*w*, 837*m*, 772*w*, 735*m*, 701*m*; ^1^H-NMR (DMSO-*d*_6_), δ: 8.48 (d, *J* = 8.3 Hz, 1H), 7.96–8.07 (m, 2H), 7.81 (ddd, *J* = 8.5 Hz, *J* = 6.8 Hz, *J* = 1.5 Hz, 1H), 7.57–7.71 (m, 2H), 3.61–3.69 (m, 2H), 3.22–3.35 (m, 2H), 1.38–1.73 (m, 6H); ^13^C-NMR (DMSO-*d*_6_), δ: 166.63, 154.67, 146.14, 137.34, 130.26, 128.99, 128.00, 127.46, 127.38, 120.04, 47.45, 42.22, 25.97, 25.31, 23.97; HR-MS: for C_15_H_17_N_2_O [M+H]^+^ calculated 241.1335 *m/z*, found 241.1384 *m/z*.

*N-Cyclopentylquinoline-2-carboxamide* (**5**). Yield 36%; Mp. 63–64 °C; IR (Zn/Se ATR, cm^−1^): 3,387*m*, 3,305*m*, 2,933*m*, 2,848*m*, 1,655*s*, 1,614*w*, 1,561*w*, 1,524*s*, 1,497*s*, 1,446*m*, 1,373*w*, 1,205*w*, 1,160*m*, 1,136*m*, 1,074*w*, 849*m*, 796*w*, 779*s*, 735*w*; ^1^H-NMR (DMSO-*d*_6_), δ: 8.68 (d, *J* = 8.0 Hz, 1H), 8.54 (d, *J* = 8.5 Hz, 1H), 8.15 (dd, *J* = 8.4 Hz, *J* = 2.6 Hz, 2H), 8.06 (d, *J* = 8.0 Hz, 1H), 7.85 (dd, *J* = 8.3 Hz, *J* = 7.0 Hz, 1H), 7.65–7.74 (m, 1H), 4.30 (sxt, *J* = 7.3 Hz, 1H), 1.85–2.01 (m, 2H), 1.48–1.74 (m, 6H); ^13^C-NMR (DMSO‑*d*_6_), δ: 163.59, 150.30, 145.97, 137.85, 130.46, 129.22, 128.77, 128.09, 128.00, 118.66, 50.72, 32.17, 23.60; HR-MS: for C_15_H_17_N_2_O [M+H]^+^ calculated 241.1335 *m/z*, found 241.1375 *m/z*.

*N-Cyclohexylquinoline-2-carboxamide* (**6**) [40]. Yield 40%; Mp. 98–99 °C; IR (Zn/Se ATR, cm^−1^): 3,329*m*, 3,313*m*, 2,969*s*, 2,933*m*, 2,875*m*, 1,648*s*, 1,556*w*, 1,522*s*, 1,498*s*, 1,470*m*, 1,423*s*, 1,382*w*, 1,361*m*, 1,341*w*, 1,324*w*, 1,201*w*, 1,178*m*, 1,130*m*, 1,105*w*, 903*w*, 844*m*, 776*w*, 735*w*, 674*w*; ^1^H-NMR (DMSO-*d*_6_), δ: 8.57 (d, *J* = 8.5 Hz, 1H), 8.54 (d, *J* = 8.5 Hz, 1H), 8.16 (d, *J* = 8.3 Hz, 2H), 8.06 (d, *J* = 8.0 Hz, 1H), 7.81–7.88 (m, 1H), 7.66–7.72 (m, 1H), 3.78–3.89 (m, 1H), 1.06–1.88 (m, 10H); ^13^C-NMR (DMSO-*d*_6_), δ: 162.92, 150.27, 145.94, 137.84, 130.44, 129.20, 128.77, 128.06, 127.98, 118.64, 48.12, 32.26, 25.12, 24.83; HR-MS: for C_16_H_19_N_2_O [M+H]^+^ calculated 255.1492 *m/z*, found 255.1525 *m/z*.

*N-Cycloheptylquinoline-2-carboxamide* (**7**). Yield 39%; Mp. 72–73 °C; IR (Zn/Se ATR, cm^−1^): 3,056*w*, 2,958*m*, 2,864*m*, 1,615*s*, 1,552*m*, 1,469*m*, 1,436*m*, 1,417*s*, 1,373*w*, 1,332*w*, 1,205*w*, 1,181*w*, 1,156*w*, 842*m*, 764*m*, 731*w*, 657*w*; ^1^H-NMR (DMSO-*d*_6_), δ: 8.61 (d, *J* = 8.5 Hz, 1H), 8.54 (d, *J* = 8.3 Hz, 1H), 8.15 (d, *J* = 8.5 Hz, 2H), 8.06 (d, *J* = 7.5 Hz, 1H), 7.85 (ddd, *J* = 8.4 Hz, *J* = 6.9 Hz, *J* = 1.3 Hz, 1H), 7.69 (ddd, *J* = 8.0 Hz, *J* = 6.9 Hz, *J* = 1.1 Hz, 1H), 4.01 (qt, *J* = 9.0 Hz, *J* = 4.5 Hz, 1H), 1.81–1.93 (m, 2H), 1.37–1.72 (m, 10H); ^13^C-NMR (DMSO-*d*_6_), δ: 162.64, 150.30, 145.94, 137.85, 130.45, 129.21, 128.78, 128.06, 127.98, 118.63, 50.32, 34.30, 27.57, 23.88; HR-MS: for C_17_H_21_N_2_O [M+H]^+^ calculated 269.1648 *m/z*, found 269.1619 *m/z*.

*N-Cyclooctylquinoline-2-carboxamide* (**8**) [41]. Yield 44%; Mp. 72–73 °C; IR (Zn/Se ATR, cm^−1^): 3,301*w*, 2,954*m*, 2,916*s*, 2,848*s*, 1,667*s*, 1,649*s*, 1,593*w*, 1,532*s*, 1,498*s*, 1,462*m*, 1,418*m*, 1,373*w*, 1,340*w*, 1,315*w*, 1,295*m*, 1,209*w*, 1,168*m*, 1136*w*, 1,111*w*, 903*w*, 850*s*, 796*w*, 772*s*, 735*m*, 715*w*, 666*w*; ^1^H-NMR (DMSO-*d*_6_), δ: 8.59 (d, *J* = 7.8 Hz, 1H), 8.55 (d, *J* = 8.5 Hz, 1H), 8.15 (d, *J* = 8.3 Hz, 2H), 8.06 (d, *J* = 8.0 Hz, 1H), 7.81–7.88 (m, 1H), 7.66–7.73 (m, 1H), 4.00–4.12 (m, 1H), 1.45–1.81 (m, 14H); ^13^C-NMR (DMSO-*d*_6_), δ: 162.60, 150.32, 145.94, 137.85, 130.45, 129.21, 128.78, 128.06, 127.98, 118.61, 49.11, 31.80, 26.71, 25.00, 23.50; HR-MS: for C_18_H_23_N_2_O [M+H]^+^ calculated 283.1805 *m/z*, found 283.1777 *m/z*.

*N-Phenylquinoline-2-carboxamide* (**9**). Yield 59%; Mp. 139–140 °C (Mp. 139.5–140 °C [42]); IR (Zn/Se ATR, cm^−1^): 3,293*m*, 2,927*s*, 2,855*m*, 1,643*s*, 1,562*m*, 1,530*s*, 1,500*s*, 1,426*m*, 1,315*w*, 1,205*w*, 1,185*w*, 1,160*w*, 1,070*w*, 1,042*w*, 910*m*, 870*w*, 841*m*, 776*m*, 731*w*; ^1^H-NMR (DMSO-*d*_6_), δ: 10.75 (bs, 1H), 8.62 (d, *J* = 8.5 Hz, 1H), 8.22–8.30 (m, 2H), 8.11 (d, *J* = 7.5 Hz, 1H), 7.94–7.99 (m, 2H), 7.91 (ddd, *J* = 8.5 Hz, *J* = 7.0 Hz, *J* = 1.4 Hz, 1H), 7.75 (ddd, *J* = 8.1 Hz, *J* = 7.0 Hz, *J* = 1.3 Hz, 1H), 7.38–7.45 (m, 2H), 7.12–7.19 (m, 1H); ^13^C-NMR (DMSO-*d*_6_), δ: 162.71, 150.08, 145.88, 138.31, 138.22, 130.68, 129.35, 128.94, 128.78, 128.37, 128.15, 124.05, 120.29, 118.77; HR-MS: for C_16_H_13_N_2_O [M+H]^+^ calculated 249.1022 *m/z*, found 249.1015 *m/z*.

*N-Benzylquinoline-2-carboxamide* (**10**). Yield 60%; Mp. 123–124 °C (Mp. 124–125 °C [43]); IR (Zn/Se ATR, cm^−1^): 3,273*m*, 2,952*m*, 2,856*m*, 1,657*s*, 1,643*s*, 1,589*w*, 1,562*m*, 1,527*s*, 1,498*s*, 1,446*w*, 1,424*m*, 1,315*w*, 1,299*w*, 1,205*m*, 1,181 *w*, 1,152*w*, 1,143*w*, 956*w*, 899*m*, 846*s*, 792*m*, 770*m*, 776*m*, 739*m*, 686*m*; ^1^H-NMR (DMSO-*d*_6_), δ: 9.50 (t, *J* = 6.4 Hz, 1H), 8.56 (d, *J* = 8.3 Hz, 1H), 8.19 (d, *J* = 8.5 Hz, 1H), 8.14 (d, *J* = 8.5 Hz, 1H), 8.07 (dd, *J* = 8.3 Hz, *J* = 0.8 Hz, 1H), 7.86 (ddd, *J* = 8.4 Hz, *J* = 6.9 Hz, *J* = 1.5 Hz, 1H), 7.70 (ddd, *J* = 8.2 Hz, *J* = 6.9 Hz, *J* = 1.0 Hz, 1H), 7.36–7.41 (m, 2 H), 7.29–7.35 (m, 2H), 7.20–7.26 (m, 1H), 4.59 (d, *J* = 6.5 Hz, 2H); ^13^C-NMR (DMSO-*d*_6_), δ: 164.13, 150.15, 146.04, 139.52, 137.88, 130.52, 129.19, 128.84, 128.30, 128.11, 128.07, 127.46, 126.82, 118.74, 42.56; HR-MS: for C_17_H_15_N_2_O [M+H]^+^ calculated 263.1179 *m/z*, found 263.1202 *m/z*.

*N-(2-Phenylethyl)quinoline-2-carboxamide* (**11**) [44]. Yield 38%; Mp. 80–81 °C; IR (Zn/Se ATR, cm^−1^): 3,236*w*, 2,935*s*, 2,852*m*, 1,633*s*, 1,552*w*, 1,467*m*, 1,450*m*, 1,422*m*, 1,344*w*, 1,279*m*, 1,103*w*, 1,025*w*, 1,005*w*, 952*w*, 890*w*, 841*m*, 760*m*, 731*w*, 682*w*; ^1^H-NMR (DMSO-*d*_6_), δ: 9.00 (t, *J* = 5.90 Hz, 1H), 8.54 (d, *J* = 8.5 Hz, 1H), 8.17 (d, *J* = 8.5 Hz, 1H), 8.11 (d, *J* = 8.5 Hz, 1H), 8.06 (d, *J* = 8.0 Hz, 1H), 7.85 (td, *J* = 7.7 Hz, *J* = 1.3 Hz, 1H), 7.66–7.73 (m, 1H), 7.25–7.33 (m, 4H), 7.16–7.22 (m, 1H), 3.58–3.65 (m, 2H), 2.92 (t, *J* = 7.5 Hz, 2H); ^13^C-NMR (DMSO-*d*_6_), δ: 163.88, 150.14, 145.96, 139.36, 137.83, 130.48, 129.13, 128.78, 128.64, 128.38, 128.08, 128.00, 126.14, 118.59, 40.58, 35.23; HR-MS: for C_18_H_17_N_2_O [M+H]^+^ calculated 277.1335 *m/z*, found 277.1384 *m/z*.

*N-(2-Hydroxyphenyl)quinoline-2-carboxamide* (**12a**) [45]. Yield 27%; Mp. 219–220 °C; ^1^H-NMR (DMSO-*d*_6_), δ: 10.67 (bs, 1H), 10.44 (bs, 1H), 8.63 (d, *J* = 8.5 Hz, 1H), 8.44 (d, *J* = 7.8 Hz, 1H), 8.29 (d, *J* = 8.3 Hz, 1H), 8.15 (d, *J* = 8.3 Hz, 1H), 8.10 (d, *J* = 8.0 Hz, 1H), 7.88 (t, *J* = 7.3 Hz, 1H), 7.73 (t, *J* = 7.2 Hz, 1H), 7.00 (m, 2H), 6.89 (m, 1H); ^13^C-NMR (DMSO-*d*_6_), δ: 161.26, 149.65, 146.77, 145.74, 138.59, 130.91, 129.29, 129.08, 128.46, 128.22, 126.20, 124.29, 119.37, 119.26, 118.42, 114.84; HR-MS: for C_16_H_13_N_2_O_2_ [M+H]^+^ calculated 265.0977 *m/z*, found 265.0983 *m/z*.

*N-(3-Hydroxyphenyl)quinoline-2-carboxamide* (**12b**). Yield 52%; Mp. 154–155 °C; IR (Zn/Se ATR, cm^−1^): 3,338*w*, 2,973*w*, 1,760*m*, 1,680*m*, 1,605*w*, 1,530*s*, 1,504*s*, 1,462*w*, 1,425*m*, 1,263*m*, 1,235*m*, 1,216*m*, 1,171*w*, 1,145*s*, 1,096*m*, 1,054*w*, 1,002*w*, 964*w*, 908*m*, 879*w*, 841*m*, 773*s*, 754*s*, 685*m*, 665*m*; ^1^H-NMR (DMSO-*d*_6_), δ: 10.98 (bs, 1H), 8.62 (d, *J* = 8.7 Hz, 1H), 8.20–8.36 (m, 3H), 8.06–8.18 (m, 1H), 7.87–7.97 (m, 2H), 7.71–7.83 (m, 2H), 7.54 (t, *J* = 8.2 Hz, 1H), 7.18 (dd, *J* = 8.0 Hz, *J* = 1.8 Hz, 1H); ^13^C-NMR (DMSO-*d*_6_), δ: 163.05, 150.91, 149.86, 147.01, 145.90, 139.63, 138.29, 130.94, 129.99, 129.19, 128.49, 128.16, 121.35, 118.83, 117.99, 113.65; HR-MS: for C_16_H_13_N_2_O_2_ [M+H]^+^ calculated 265.0977 *m/z*, found 265.0985 *m/z*.

*N-(4-Hydroxyphenyl)quinoline-2-carboxamide* (**12c**) [46]. Yield 48%; Mp. 230–231 °C; ^1^H-NMR (DMSO-*d*_6_), δ: 10.34 (bs, 1H), 8.28–8.48 (m, 3H), 8.21 (d, *J* = 8.5 Hz, 1H), 7.88–8.04 (m, 3H), 7.77–7.87 (m, 1H), 7.69 (m, 1H), 7.39 (d, *J* = 9.0 Hz, 2H); ^13^C-NMR (DMSO-*d*_6_), δ: 162.11, 149.40, 147.23, 146.23, 137.94, 135.82, 130.84, 129.62, 129.45, 128.95, 127.60, 122.38, 120.66, 118.73; HR-MS: for C_16_H_13_N_2_O_2_ [M+H]^+^ calculated 265.0977 *m/z*, found 265.0971 *m/z*.

*N-(2-Methoxyphenyl)quinoline-2-carboxamide* (**13a**) [47]. Yield 37%; Mp. 111–112 °C; IR (Zn/Se ATR, cm^−1^): 3,382*w*, 1,676*s*, 1,596*m*, 1,532*s*, 1,485*w*, 1,454*m*, 1,426*m*, 1,334*w*, 1,288*w*, 1,253*m*, 1,138*m*, 1,129*m*, 1,093*w*, 1,020*s*, 951*w*, 908*m*, 873*w*, 840*m*, 820*w*, 770*s*, 732*s*; ^1^H-NMR (DMSO-*d*_6_), δ: 10.68 (bs, 1H), 8.59 (d, *J* = 8.5 Hz, 1H), 8.49 (d, *J* = 7.8 Hz, 1H), 8.25 (d, *J* = 8.5 Hz, 1H), 8.15 (d, *J* = 8.5 Hz, 1H), 8.07 (d, *J* = 8.3 Hz, 1H), 7.87 (t, *J* = 7.3 Hz, 1H), 7.67–7.75 (m, 1H), 7.11 (d, *J* = 4.0 Hz, 2H), 7.01 (dt, *J* = 8.2 Hz, *J* = 4.2 Hz, 1H), 3.98 (s, 3H); ^13^C-NMR (DMSO-*d*_6_), δ: 161.25, 149.34, 148.51, 145.62, 138.55, 130.82, 129.30, 129.06, 128.44, 128.14, 126.87, 124.25, 120.68, 118.84, 118.27, 110.91, 56.05; HR-MS: for C_17_H_15_N_2_O_2_ [M+H]^+^ calculated 279.1134 *m/z*, found 279.1148 *m/z*.

*N-(3-Methoxyphenyl)quinoline-2-carboxamide* (**13b**). Yield 47%; Mp. 117–118 °C; IR (Zn/Se ATR, cm^−1^): 3,352*w*, 1,687*m*, 1,589*m*, 1,524*m*, 1,503*m*, 1,456*m*, 1,425 *m*, 1,334*w*, 1,284*m*, 1,203 *m*, 1,157*m*, 1,128*m*, 1,049*s*, 906*w*, 876*m*, 854 *m*, 823 *w*, 798*w*, 762*s*, 740*s*, 685*m*; ^1^H-NMR (DMSO-*d*_6_), δ: 10.73 (bs, 1H), 8.58 (d, *J* = 8.5 Hz, 1H), 8.19–8.32 (m, 2H), 8.07 (d, *J* = 8.0 Hz, 1H), 7.82–7.96 (m, 1H), 7.65–7.79 (m, 2H), 7.59 (dd, *J* = 8.0 Hz, *J* = 1.0 Hz, 1H), 7.29 (t, *J* = 8.2 Hz, 1H), 6.72 (dd, *J* = 8.3 Hz, *J* = 2.01 Hz, 1H), 3.78 (s, 3H); ^13^C-NMR (DMSO-*d*_6_), δ: 162.70, 159.61, 149.99, 145.88, 139.53, 138.23, 130.67, 129.61, 129.37, 128.97, 128.37, 128.16, 118.75, 112.47, 109.68, 105.91, 55.09; HR-MS: for C_17_H_15_N_2_O_2_ [M+H]^+^ calculated 279.1134 *m/z*, found 279.1129 *m/z*.

*N-(4-Methoxyphenyl)quinoline-2-carboxamide* (**13c**) [48]. Yield 53%; Mp. 130–131 °C; ^1^H-NMR (DMSO-*d*_6_), δ: 10.65 (bs, 1H), 8.57 (d, *J* = 8.5 Hz, 1H), 8.24 (d, *J* = 8.5 Hz, 2H), 8.07 (d, *J* = 7.8 Hz, 1H), 7.82–7.95 (m, 3H), 7.63–7.78 (m, 1H), 6.97 (d, *J* = 9.0 Hz, 2H), 3.75 (s, 3H); ^13^C-NMR (DMSO-*d*_6_), δ: 162.28, 155.80, 150.25, 145.90, 138.09, 131.47, 130.59, 129.32, 128.87, 128.22, 128.11, 121.85, 118.73, 113.87, 55.17; HR-MS: for C_17_H_15_N_2_O_2_ [M+H]^+^ calculated 279.1134 *m/z*, found 279.1145 *m/z*.

*N-(2-Methylphenyl)quinoline-2-carboxamide* (**14a**). Yield 40%; Mp. 100–101 °C; IR (Zn/Se ATR, cm^−1^): 3,334*w*, 1,686*s*, 1,587*s*, 1,528*s*, 1,498*m*, 1,454*s*, 1,427*s*, 1,422 *m*, 1,373*w*, 1,305*m*, 1,249*w*, 1,201*w*, 1,132*m*, 1,091*w*, 1,040*w*, 1,013*w*, 981*w*, 954*m*, 932*w*, 907*m*, 872*m*, 842*s*, 793*w*, 765*s*, 750*s*, 731*s*, 681*s*; ^1^H-NMR (DMSO-*d*_6_), δ: 10.45 (bs, 1H), 8.60 (d, *J* = 8.5 Hz, 1H), 8.24 (d, *J* = 8.5 Hz, 1H), 8.17 (d, *J* = 8.3 Hz, 1H), 8.08 (d, *J* = 8.0 Hz, 1H), 7.95 (d, *J* = 7.8 Hz, 1H), 7.83–7.91 (m, 1H), 7.69–7.77 (m, 1H), 7.22–7.31 (m, 2H), 7.08–7.16 (m, 1H), 2.37 (s, 3H); ^13^C-NMR (DMSO-*d*_6_), δ: 161.92, 149.67, 145.74, 138.35, 135.97, 130.72, 130.42, 130.01, 129.38, 129.01, 128.38, 128.11, 126.40, 124.96, 122.65, 118.49, 17.49; HR-MS: for C_17_H_15_N_2_O [M+H]^+^ calculated 263.1184 *m/z*, found 263.1182 *m/z*.

*N-(3-Methylphenyl)quinoline-2-carboxamide* (**14b**). Yield 43%; Mp. 82–83 °C; IR (Zn/Se ATR, cm^−1^): 3,355*w*, 1,685*m*, 1,592 *m*, 1,527*s*, 1,503*s* 1,457*w*, 1,424 *m*, 1,300*w*, 1,171*w*, 1,125*m*, 908*w*, 852*m*, 773*s*, 740*w*, 690*s*; ^1^H-NMR (DMSO-*d*_6_), δ: 10.66 (bs, 1H), 8.61 (d, *J* = 8.5 Hz, 1H), 8.25 (dd, *J* = 7.9 Hz, *J* = 5.40 Hz, 2H), 8.10 (d, *J* = 8.0 Hz, 1H), 7.90 (t, *J* = 7.5 Hz, 1H), 7.67–7.84 (m, 3H), 7.27 (t, *J* = 7.7 Hz, 1H), 6.96 (d, *J* = 7.3 Hz, 1H), 2.32 (s, 3H); ^13^C-NMR (DMSO-*d*_6_), δ: 162.54, 150.03, 145.88, 138.22, 138.20, 138.03, 130.68, 129.35, 128.94, 128.65, 128.35, 128.15, 124.75, 120.72, 118.70, 117.35, 21.23; HR-MS: for C_17_H_15_N_2_O [M+H]^+^ calculated 263.1184 *m/z*, found 263.1191 *m/z*.

*N-(4-Methylphenyl)quinoline-2-carboxamide* (**14c**). Yield 44%; Mp. 107–108 °C (Mp. 109.5–110 °C [39]); ^1^H-NMR (DMSO-*d*_6_), δ: 10.67 (bs, 1H), 8.59 (d, *J* = 8.5 Hz, 1H), 8.18–8.30 (m, 2H), 8.08 (d, *J* = 8.0 Hz, 1H), 7.79–7.94 (m, 3H), 7.72 (t, *J* = 7.4 Hz, 1H), 7.18 (d, *J* = 8.3 Hz, 2H), 2.27 (s, 3H); ^13^C-NMR (DMSO-*d*_6_), δ: 162.51, 150.17, 145.93, 138.21, 135.86, 133.09, 130.69, 129.39, 129.24, 128.94, 128.35, 128.18, 120.27, 118.77, 20.59; HR‑MS: for C_17_H_15_N_2_O [M+H]^+^ calculated 263.1184 *m/z*, found 263.1193 *m/z*.

*N-(2-Fluorophenyl)quinoline-2-carboxamide* (**15a**). Yield 30%; Mp. 116–117 °C; IR (Zn/Se ATR, cm^−1^): 3,328*w*, 1,691*m*, 1,615*m*, 1,591*w*, 1,530*s*, 1,504*m*, 1,477*w*, 1,454*m*, 1,428*m*, 1,317*w*, 1,247*w*, 1,185*w*, 1,126*m*, 1,088*w*, 910*w*, 837*m*, 772*s*, 746*s*, 683*m*; ^1^H-NMR (DMSO-*d*_6_), δ: 10.48 (bs, 1H), 8.57 (d, *J* = 8.5 Hz, 1H), 8.17–8.25 (m, 2H), 8.13 (d, *J* = 8.5 Hz, 1H), 8.05 (d, *J* = 8.0 Hz, 1H), 7.85 (t, *J* = 7.3 Hz, 1H), 7.65–7.76 (m, 1H), 7.28–7.40 (m, 1H), 7.13–7.27 (m, 2H); ^13^C-NMR (DMSO-*d*_6_), δ: 162.00, 153.58 (d, ^1^*J*_FC_ = 244 Hz), 148.95, 145.67, 138.39, 130.76, 129.24, 129.15 (d, ^2^*J*_FC_ = 19.1 Hz), 128.45, 128.08, 125.70 (d, ^3^*J*_FC_ = 11.0 Hz), 125.53 (d, ^3^*J*_FC_ = 7.3 Hz), 124.63 (d, ^4^*J*_FC_ = 3.7 Hz), 122.91, 118.37, 115.43 (d, ^2^*J*_FC_ = 19.1 Hz); HR-MS: for C_16_H_12_FN_2_O [M+H]^+^ calculated 267.0934 *m/z*, found 267.0950 *m/z*.

*N-(3-Fluorophenyl)quinoline-2-carboxamide* (**15b**). Yield 38%; Mp. 126–127 °C; IR (Zn/Se ATR, cm^−1^): 3,343*w*, 1,690*s*, 1,588*m*, 1,531*s*, 1,504*m*, 1,481*s*, 1,409*s*, 1,170*m*, 1,137*m*, 899*m*, 841*s*, 791*m*, 768*s*, 738*m*, 682*s*; ^1^H-NMR (DMSO-*d*_6_), δ: 10.91 (bs, 1H), 8.58 (d, *J* = 8.5 Hz, 1H), 8.16–8.31 (m, 2H), 8.08 (d, *J* = 8.3 Hz, 1H), 7.95 (d, *J* = 11.8 Hz, 1H), 7.86–7.92 (m, 1H), 7.79 (d, *J* = 8.3 Hz, 1H), 7.68–7.75 (m, 1H), 7.35–7.49 (m, 1H), 6.96 (td, *J* = 8.4 Hz, *J* = 2.0 Hz, 1H); ^13^C-NMR (DMSO-*d*_6_), δ: 163.01, 162.15 (d, ^1^*J*_FC_ = 241 Hz), 149.73, 145.86, 140.11 (d, ^3^*J*_FC_ = 11.0 Hz), 138.18, 130.65, 130.31 (d, ^3^*J*_FC_ = 9.5 Hz), 129.33, 128.97, 128.39, 128.12, 118.77, 116.15 (d, ^4^*J*_FC_ = 2.9 Hz), 110.47 (d, ^2^*J*_FC_ = 21.3 Hz), 107.09 (d, ^2^*J*_FC_ = 26.4 Hz); HR-MS: for C_16_H_12_FN_2_O [M+H]^+^ calculated 267.0934 *m/z*, found 267.0953 *m/z*.

*N-(4-Fluorophenyl)quinoline-2-carboxamide* (**15c**) [46,48]. Yield 33%; Mp. 115–116 °C; ^1^H-NMR (DMSO-*d*_6_), δ: 10.83 (bs, 1H), 8.57 (d, *J* = 8.3 Hz, 1H), 8.17–8.29 (m, 2H), 8.06 (d, *J* = 8.0 Hz, 1H), 7.94–8.02 (m, 2H), 7.87 (td, *J* = 7.7 Hz, *J* = 1.3 Hz, 1H), 7.66–7.76 (m, 1H), 7.17–7.28 (m, 2H); ^13^C-NMR (DMSO-*d*_6_), δ: 162.76, 158.58 (d, ^1^*J*_FC_ = 237 Hz), 150.03, 145.95, 138.18, 134.81 (d, ^4^*J*_FC_ = 2.2 Hz), 130.67, 129.39, 128.98, 128.37, 128.17, 122.31 (d, ^3^*J*_FC_ = 7.3 Hz), 118.83, 115.26 (d, ^2^*J*_FC_ = 22.7 Hz); HR-MS: for C_16_H_12_FN_2_O [M+H]^+^ calculated 267.0934 *m/z*, found 267.0954 *m/z*.

*N-(2-Chlorophenyl)quinoline-2-carboxamide* (**16a**) [48]. Yield 39%; Mp. 130–131 °C; ^1^H-NMR (DMSO-*d*_6_), δ: 10.77 (bs, 1H), 8.58 (d, *J* = 8.5 Hz, 1H), 8.43 (d, *J* = 8.0 Hz, 1H), 8.21 (d, *J* = 8.5 Hz, 1H), 8.10 (d, *J* = 8.5 Hz, 1H), 8.05 (d, *J* = 8.3 Hz, 1H), 7.85 (t, *J* = 7.5 Hz, 1H), 7.64–7.75 (m, 1H), 7.54 (d, *J* = 7.8 Hz, 1H), 7.39 (t, *J* = 7.7 Hz, 1H), 7.10–7.24 (m, 1H); ^13^C-NMR (DMSO-*d*_6_), δ: 161.54, 148.70, 145.47, 138.50, 134.21, 130.75, 129.29, 129.20, 129.07, 128.46, 128.00, 127.88, 125.23, 123.38, 121.27, 118.15; HR-MS: for C_16_H_12_ClN_2_O [M+H]^+^ calculated 283.0638 *m/z*, found 283.0652 *m/z*.

*N-(3-Chlorophenyl)quinoline-2-carboxamide* (**16b**) [48]. Yield 46%; Mp. 127–128 °C; ^1^H-NMR (DMSO-*d*_6_), δ: 10.90 (bs, 1H), 8.58 (d, *J* = 8.5 Hz, 1H), 8.18–8.31 (m, 2H), 8.15 (s, 1H), 8.07 (d, *J* = 8.0 Hz, 1H), 7.82–7.97 (m, 2H), 7.66–7.78 (m, 1H), 7.40 (t, *J* = 8.0 Hz, 1H), 7.11–7.23 (m, 1H); ^13^C-NMR (DMSO-*d*_6_), δ: 163.02, 149.67, 145.87, 139.85, 138.20, 133.12, 130.68, 130.36, 129.34, 128.98, 128.43, 128.14, 123.70, 119.82, 118.77; HR-MS: for C_16_H_12_ClN_2_O [M+H]^+^ calculated 283.0638 *m/z*, found 283.0648 *m/z*.

*N-(4-Chlorophenyl)quinoline-2-carboxamide* (**16c**). Yield 34%; Mp. 134–135 °C (Mp. 135–135.5 °C [39]); ^1^H-NMR (DMSO-*d*_6_), δ: 10.88 (bs, 1H), 8.58 (d, *J* = 8.5 Hz, 1H), 8.17–8.30 (m, 2H), 8.08 (d, *J* = 8.0 Hz, 1H), 8.01 (d, *J* = 8.8 Hz, 2H), 7.84–7.93 (m, 1H), 7.68–7.77 (m, 1H), 7.43 (d, *J* = 8.8 Hz, 2H); ^13^C-NMR (DMSO-*d*_6_), δ: 162.87, 149.85, 145.88, 138.16, 137.34, 130.65, 129.34, 128.95, 128.62, 128.37, 128.14, 127.69, 121.92, 118.78; HR-MS: for C_16_H_12_ClN_2_O [M+H]^+^ calculated 283.0638 *m/z*, found 283.0631 *m/z*.

*N-(2-Bromophenyl)quinoline-2-carboxamide* (**17a**). Yield 26%; Mp. 134–135 °C; IR (Zn/Se ATR, cm^−1^): 3,277*w*, 1,689*s*, 1,588*m*, 1,579*m*, 1,543*m*, 1,530*s*, 1,496*m*, 1,440*m*, 1,427*m*, 1,302*w*, 1,132*w*, 1,204*m*, 908*w*, 842*m*, 768*s*, 736*m*, 698*m*; ^1^H-NMR (DMSO-*d*_6_), δ: 10.82 (bs, 1H), 8.60 (d, *J* = 8.5 Hz, 1H), 8.44 (d, *J* = 8.3 Hz, 1H), 8.23 (d, *J* = 8.5 Hz, 1H), 8.13 (d, *J* = 8.5 Hz, 1H), 8.07 (d, *J* = 8.3 Hz, 1H), 7.87 (t, *J* = 7.5 Hz, 1H), 7.64–7.77 (m, 2H), 7.44 (t, *J* = 7.8 Hz, 1H), 7.10 (t, *J* = 7.7 Hz, 1H); ^13^C-NMR (DMSO-*d*_6_), δ: 161.61, 148.71, 145.50, 138.60, 135.46, 132.58, 130.83, 129.26, 129.13, 128.55, 128.53, 128.08, 125.74, 121.39, 118.19, 114.08; HR-MS: for C_16_H_12_BrN_2_O [M+H]^+^ calculated 327.0133 *m/z*, found 327.0138 *m/z*.

*N-(3-Bromophenyl)quinoline-2-carboxamide* (**17b**). Yield 35%; Mp. 139–140 °C; IR (Zn/Se ATR, cm^−1^): 3,318*w*, 1,687*m*, 1,581*m*, 1,519*m*, 1,478*w*, 1,408*m*, 1,296*w*, 1,124*m*, 1,067*w*, 912*w*, 847*m*, 764*s*, 685*m*; ^1^H-NMR (DMSO-*d*_6_), δ: 10.89 (bs, 1H), 8.60 (d, *J* = 8.3 Hz, 1H), 8.19-8.32 (m, 3H), 8.09 (d, *J* = 8.0 Hz, 1H), 7.96 (d, *J* = 7.5 Hz, 1H), 7.87–7.93 (m, 1H), 7.68–7.78 (m, 1H), 7.27–7.40 (m, 2H); ^13^C-NMR (DMSO-*d*_6_), δ: 163.02, 149.67, 145.86, 139.98, 138.21, 130.69, 130.67, 129.32, 128.97, 128.44, 128.14, 126.59, 122.66, 121.55, 119.14, 118.77; HR-MS: for C_16_H_12_BrN_2_O [M+H]^+^ calculated 327.0133 *m/z*, found 327.0143 *m/z*.

*N-(4-Bromophenyl)quinoline-2-carboxamide* (**17c**). Yield 57%; Mp. 157–158 °C; IR (Zn/Se ATR, cm^−1^): 3,355*w*, 1,693*s*, 1,581*m*, 1,522*s*, 1,496*s*, 1,423*w*, 1,389*m*, 1,305*w*, 1,120*m*, 1,095*w*, 1,068*m*, 998*w*, 907*w*, 839*s*, 807*s*, 769*s*, 693*w*; ^1^H-NMR (DMSO-*d*_6_), δ: 10.84 (bs, 1H), 8.58 (d, *J* = 8.5 Hz, 1H), 8.18–8.30 (m, 2H), 8.07 (d, *J* = 8.3 Hz, 1H), 7.95 (d, *J* = 8.8 Hz, 2H), 7.86–7.92 (m, 1H), 7.67–7.78 (m, 1H), 7.56 (d, *J* = 8.8 Hz, 2H); ^13^C-NMR (DMSO-*d*_6_), δ: 162.86, 149.82, 145.87, 138.16, 137.74, 131.53, 130.64, 129.33, 128.94, 128.37, 128.12, 122.28, 118.77, 115.80; HR-MS: for C_16_H_12_BrN_2_O [M+H]^+^ calculated 327.0133 *m/z*, found 327.0129 *m/z*.

*N-(2-Trifluoromethylphenyl)quinoline-2-carboxamide* (**18a**). Yield 32%; Mp. 120–121 °C; IR (Zn/Se ATR, cm^−1^): 3,316*w*, 1,698*s*, 1,590*s*, 1,537*s*, 1,498*w*, 1,452*m*, 1,423*m*, 1,320*m*, 1,288*m*, 1,244*w*, 1,202*w*, 1,165*m*, 1,124*m*, 1,094*m*, 1,054*m*, 1,026*m*, 953*w*, 906*w*, 871*w*, 836*m*, 792*w*, 763*s*, 676*m*; ^1^H-NMR (DMSO-*d*_6_), δ: 10.78 (bs, 1H), 8.61 (d, *J* = 8.3 Hz, 1H), 8.36 (d, *J* = 8.3 Hz, 1H), 8.23 (d, *J* = 8.3 Hz, 1H), 8.07 (t, *J* = 8.3 Hz, 2H), 7.87 (t, *J* = 7.5 Hz, 1H), 7.64–7.81 (m, 3H), 7.38 (t, *J* = 7.7 Hz, 1H); ^13^C-NMR (DMSO-*d*_6_), δ: 162.05, 148.48, 145.53, 138.74, 135.14, 133.57, 131.01, 129.48 (q, ^2^*J*_FC_ = 37 Hz), 129.21, 129.17, 128.68, 128.16, 126.41 (q, ^3^*J*_FC_ = 5.1 Hz), 125.05, 124.10 (q, ^1^*J*_FC_ = 274 Hz), 123.89 (q, ^3^*J*_FC_ = 5.9 Hz), 118.31; HR-MS: for C_17_H_12_F_3_N_2_O [M+H]^+^ calculated 317.0896 *m/z*, found 317.0891 *m/z*.

*N-(3-Trifluoromethylphenyl)quinoline-2-carboxamide* (**18b**). Yield 31%; Mp. 121–122 °C; IR (Zn/Se ATR, cm^−1^): 3,339*w*, 1,692*s*, 1,614*w*, 1,536*m*, 1,490*m*, 1,424*w*, 1,330*s*, 1,223*w*, 1,166*m*, 1,109*s*, 1,091*s*, 1,065*m*, 952*w*, 933*w*, 874*s*, 844*m*, 808*s*, 771*s*, 744*w*, 698*s*; ^1^H-NMR (DMSO-*d*_6_), δ: 11.08 (bs, 1H), 8.59 (d, *J* = 8.5 Hz, 1H), 8.46 (s, 1H), 8.17–8.31 (m, 3H), 8.08 (d, *J* = 8.0 Hz, 1H), 7.89 (t, *J* = 7.4 Hz, 1H), 7.68–7.78 (m, 1H), 7.61 (t, *J* = 8.0 Hz, 1H), 7.46 (d, *J* = 7.5 Hz, 1H); ^13^C-NMR (DMSO-*d*_6_), δ: 163.26, 149.64, 145.90, 139.23, 138.21, 130.69, 129.86, 129.35 (q, ^2^*J*_FC_ = 32 Hz), 129.34, 129.03, 128.45, 128.16, 124.20 (q, ^1^*J*_FC_ = 273 Hz), 123.91, 120.24 (q, ^3^*J*_FC_ = 3.7 Hz), 118.78, 116.61 (q, ^3^*J*_FC_ = 3.7 Hz); HR-MS: for C_17_H_12_F_3_N_2_O [M+H]^+^ calculated 317.0896 *m/z*, found 317.0892 *m/z*.

*N-(4-Trifluoromethylphenyl)quinoline-2-carboxamide* (**18c**) [41,44]. Yield 43%; Mp. 147–148 °C; ^1^H-NMR (DMSO-*d*_6_), δ: 11.02 (bs, 1H), 8.59 (d, *J* = 8.3 Hz, 1H), 8.26 (d, *J* = 8.5 Hz, 1H), 8.23 (d, *J* = 8.3 Hz, 1H), 8.19 (d, *J* = 8.5 Hz, 2H), 8.08 (d, *J* = 8.0 Hz, 1H), 7.86–7.93 (m, 1H), 7.69–7.77 (m, 3H); ^13^C-NMR (DMSO-*d*_6_), δ: 163.27, 149.63, 145.88, 141.95, 138.23, 130.69, 129.38, 129.03, 128.48, 128.14, 125.96 (q, ^3^*J*_FC_ = 3.7 Hz), 124.39 (q, ^1^*J*_FC_ = 271 Hz), 124.00 (q, ^2^*J*_FC_ = 32 Hz), 120.29, 118.81; HR-MS: for C_17_H_12_F_3_N_2_O [M+H]^+^ calculated 317.0896 *m/z*, found 317.0890 *m/z*.

*N-(2-Nitrophenyl)quinoline-2-carboxamide* (**19a**). Yield 29%; Mp. 181–182 °C (Mp. 179.5–180 °C [39]); ^1^H-NMR (DMSO-*d*_6_), δ: 12.55 (bs, 1H), 8.75 (d, *J* = 8.5 Hz, 1H), 8.60 (d, *J* = 8.3 Hz, 1H), 8.17–8.28 (m, 2H), 8.09 (dd, *J* = 13.9, 8.4 Hz, 2H), 7.86–7.95 (m, 1H), 7.78–7.85 (m, 1H), 7.70–7.77 (m, 1H), 7.34 (t, *J* = 7.8 Hz, 1H); ^13^C-NMR (DMSO-*d*_6_), δ: 162.61, 148.50, 145.57, 138.55, 137.44, 135.73, 133.47, 130.94, 129.20, 129.16, 128.70, 128.09, 125.80, 123.97, 121.77, 118.38; HR-MS: for C_16_H_12_N_3_O_3_ [M+H]^+^ calculated 294.0879 *m/z*, found 294.0888 *m/z*.

*N-(3-Nitrophenyl)quinoline-2-carboxamide* (**19b**). Yield 30%; Mp. 189–190 °C; IR (Zn/Se ATR, cm^−1^): 3,305*w*, 1,687*m*, 1,620*w*, 1,591*w*, 1,529*m*, 1,496*w*, 1,427*m*, 1,344*m*, 1,202*w*, 1,128*m*, 1,068*w*, 939*w*, 291*m*, 837*m*, 798*m*, 773*s*, 733*s*, 675*s*; ^1^H-NMR (DMSO-*d*_6_), δ: 11.14 (bs, 1H), 8.95 (t, *J* = 1.9 Hz, 1H), 8.57 (d, *J* = 8.5 Hz, 1H), 8.34 (dd, *J* = 8.0 Hz, *J* = 1.0 Hz, 1H), 8.16–8.28 (m, 2H), 8.06 (d, *J* = 8.0 Hz, 1H), 7.83–7.99 (m, 2H), 7.69–7.77 (m, 1H), 7.64 (t, *J* = 8.2 Hz, 1H); ^13^C-NMR (DMSO-*d*_6_), δ: 163.35, 149.40, 147.88, 145.85, 139.56, 138.16, 130.64, 129.94, 129.30, 128.99, 128.44, 128.10, 126.34, 118.74, 118.34, 114.54; HR-MS: for C_16_H_12_N_3_O_3_ [M+H]^+^ calculated 294.0879 *m/z*, found 294.0871 *m/z*.

*N-(4-Nitrophenyl)quinoline-2-carboxamide* (**19c**) [48,49]. Yield 29%; Mp. 227–228 °C; ^1^H-NMR (DMSO-*d*_6_), δ: 11.29 (bs, 1H), 8.64 (d, *J* = 8.5 Hz, 1H), 8.18–8.35 (m, 6H), 8.12 (d, *J* = 8.0 Hz, 1H), 7.88–7.99 (m, 1H), 7.78 (d, *J* = 7.8 Hz, 1H); ^13^C-NMR (DMSO-*d*_6_), δ: 163.61, 149.43, 145.88, 144.58, 142.77, 138.38, 130.83, 129.40, 129.11, 128.68, 128.21, 124.81, 120.17, 118.92; HR-MS: for C_16_H_12_N_3_O_3_ [M+H]^+^ calculated 294.0879 *m/z*, found 294.0870 *m/z*.

#### 3.2.2. General Procedure for Synthesis of Carboxamide Derivatives **20**–**38c**

2-Naphthoic acid (1 g, 5.8 mmol) was suspended in dry toluene (40 mL) and SOCl_2_ (0.6 mL, 8 mmol, 1.4 eq.) was added dropwise. The mixture was refluxed for 2 h and then evaporated to dryness. The crystalline residue was washed with dry toluene and used directly in the next step. Yield: 99%; Mp. 46–47 °C (Mp. 43 °C [50]). Into the solution of 2-naphtoyl chloride (1.1 g, 5.8 mmol) in dry CH_2_Cl_2_ (30 mL), triethylamine (1.2 mL, 0.88 g, 8.66 mmol, 1.5 eq.) and corresponding substituted amine (5.8 mmol) were added dropwise. The mixture was stirred at room temperature for 20 h after which the solvent was removed under reduced pressure. The solid residue was washed with 10% HCl and the crude product was recrystallized from EtOH, cyclohexan, CHCl_3_ or acetone. The studied compounds **20**–**38c** are presented in Table 2.

*N-Isopropyl-2-naphthamide* (**20**). Yield 18%; Mp. 174–176 °C (Mp. 170 °C [51]); IR (Zn/Se ATR, cm^−1^): 3,246*m*, 3,054*w*, 2,969*w*, 2,935*w*, 1,632*m*, 1,618s, 1,543*s* 1,499*w*, 1,352*w*, 1,327*w*, 1,294*m*, 1,201*w*, 1,168*m*, 1,136*w*, 1,106*w*, 953*w*, 911*m*, 835*m*, 781*m* 766*w*, 736*m*, 690*w*; ^1^H-NMR (DMSO-*d*_6_), δ: 8.37–8.51 (m, 2H), 7.88–8.09 (m, 4H), 7.47–7.67 (m, 2H), 4.18 (dsxt, *J* = 13.8 Hz, *J* = 6.7 Hz, 1H), 1.21 (d, *J* = 6.5 Hz, 6H); ^13^C-NMR (DMSO-*d*_6_), δ: 165.42, 134.05, 132.23, 132.16, 128.79, 127.72, 127.60, 127.44, 127.33, 126.66, 124.38, 41.14, 22.41; HR-MS: for C_14_H_16_NO [M+H]^+^ calculated 214.1226 *m/z*, found 214.1187 *m/z*.

*N-Dodecyl-2-naphthamide* (**21**). Yield 15%; Mp. 102–103 °C; IR (Zn/Se ATR, cm^−1^): 3,259*m*, 2,915*s*, 2,848*s*, 1,643*m*, 1,619*s*, 1,552*s*, 1,501*w*, 1,467*w*, 1,432*w*, 1,317*m*, 1,204*w*, 1,146*w*, 954*w*, 912*w*, 871*w*, 839*w*, 779*w*, 734*w*; ^1^H-NMR (DMSO-*d*_6_), δ: 8.61 (t, *J* = 5.8 Hz, 1H), 8.37–8.52 (m, 1H), 7.88–8.10 (m, 4H), 7.46–7.69 (m, 2H), 3.26–3.34 (m, 2H), 1.55 (quin, *J* = 7.2 Hz, 2H), 1.13–1.37 (m, 18H), 0.78-0.87 (m, 3H); ^13^C-NMR (DMSO-*d*_6_), δ: 166.06, 134.06, 132.17, 132.08, 128.78, 127.76, 127.59, 127.43, 127.28, 126.63, 124.20, 39.34, 31.32, 29.17, 29.16, 29.09, 29.06, 29.05, 28.84, 28.75, 26.55, 22.11, 13.95; HR-MS: for C_23_H_34_NO [M+H]^+^ calculated 340.2635 *m/z*, found 340.2655 *m/z*.

*1-(2-Naphthoyl)pyrrolidine* (**22**). Yield 12%; Mp. 76–77 °C (Mp. 75.5–76.5 °C [52]); IR (Zn/Se ATR, cm^−1^): 3,050*m*, 2,963*s*, 2,863*s*, 1,629*m*, 1,608*s*, 1,469*m*, 1,434*w*, 1,417*m*, 1,353*w*, 1,337*w*; ^1^H-NMR (DMSO-*d*_6_), δ: 8.10 (s, 1H), 7.97–8.03 (m, 1H), 7.91–7.97 (m, 2H), 7.51–7.64 (m, 3H), 3.51 (t, *J* = 6.9 Hz, 2H), 3.37–3.45 (m, 2H), 1.82–1.92 (m, 2H), 1.69-1.82 (m, 2H); ^13^C-NMR (DMSO-*d*_6_), δ: 168.18, 134.50, 133.23, 132.15, 128.47, 127.77, 127.60, 127.10, 126.62, 126.59, 124.61, 48.97, 46.01, 25.99, 23.97; HR-MS: for C_15_H_16_NO [M+H]^+^ calculated 226.1226 *m/z*, found 226.1200 *m/z*.

*1-(2-Naphthoyl)piperidine* (**23**) [53]. Yield 40%; Mp. 97–98 °C; IR (Zn/Se ATR, cm^−1^): 3,050*w*, 2,933*m*, 2,852*m*, 1,633*w*, 1,612*s*, 1,476*m*, 1,432*m*, 1,347*w*, 1,278*m*, 1,269*m*, 1,229*w*, 1,199*w*, 1,126*w*, 1,100*w*, 1,007*w*, 954*w*, 906*w*, 833*w*, 807*w*, 758*m*; ^1^H-NMR (DMSO-*d*_6_), δ: 7.86–8.08 (m, 4H), 7.39–7.67 (m, 4H), 3.09–3.85 (m, 4H), 1.26–1.77 (m, 6H); ^13^C-NMR (DMSO-*d*_6_), δ: 168.84, 133.91, 132.96, 132.31, 128.27, 127.98, 127.65, 126.93, 126.66, 125.96, 124.29, 48.95, 43.22, 26.51, 26.01, 24.05; HR-MS: for C_16_H_18_NO [M+H]^+^ calculated 240.1383 *m/z*, found 240.1349 *m/z*.

*N-Cyclopentyl-2-naphthamide* (**24**). Yield 21%; Mp. 190–191 °C; IR (Zn/Se ATR, cm^−1^): 3,245*s*, 2,954*m*, 2,867*w*, 1,618*m*, 1,545*m*; ^1^H-NMR (DMSO-*d*_6_), δ: 8.37–8.56 (m, 2H), 7.89–8.11 (m, 4H), 7.48–7.67 (m, 2H), 4.31 (sxt, *J* = 7.0 Hz, 1H), 1.42–2.05 (m, 8H); ^13^C-NMR (DMSO-*d*_6_), δ: 166.00, 134.04, 132.20, 132.15, 128.78, 127.68, 127.60, 127.41, 127.38, 126.62, 124.45, 51.08, 32.20, 23.70; HR-MS: for C_16_H_18_NO [M+H]^+^ calculated 240.1383 *m/z*, found 240.1372 *m/z*.

*N-Cyclohexyl-2-naphthamide* (**25**). Yield 45%; Mp. 184–185 °C (Mp. 183–184 °C [52]); IR (Zn/Se ATR, cm^−1^): 3,316*m*, 3,051*w*, 2,933*s*, 2,917*m*, 2,850*m*, 1,635*m*, 1,622*s*, 1,601*m*, 1,531*s*, 1,446*w*, 1,318*m*, 1,288*m*, 1,231*m*, 1,202*w*, 1,151*w*, 1,081*m*, 890*m*, 859*w*, 819*m*, 779*m*, 758*m*, 728*w*, 667*w*; ^1^H-NMR (DMSO-*d*_6_), δ: 8.30–8.54 (m, 2H), 7.85–8.12 (m, 4H), 7.46–7.71 (m, 2H), 3.70–3.97 (m, 1H), 1.87 (d, *J* = 9.8 Hz, 2H), 1.74 (dd, *J* = 9.8 Hz, *J* = 2.5 Hz, 2H), 1.61 (d, *J* = 12.6 Hz, 1H), 1.23–1.43 (m, 4H), 1.04–1.21 (m, 1H); ^13^C-NMR (DMSO-*d*_6_), δ: 165.42, 134.04, 132.26, 132.15, 128.78, 127.68, 127.59, 127.41, 127.34, 126.63, 124.42, 48.49, 32.50, 25.31, 25.00; HR-MS: for C_17_H_20_NO [M+H]^+^ calculated 254.1539 *m/z*, found 254.1493 *m/z*.

*N-Cycloheptyl-2-naphthamide* (**26**). Yield 18%; Mp. 157–158 °C; IR (Zn/Se ATR, cm^−1^): 3,318*m*, 2,918*m*, 2,655*m*, 1,635*m*, 1,623*s*, 1,600*w*, 1,529*s*, 1,504*m*, 1,446*w*, 1,314*m*, 1,234*w*, 1,201*w*, 1,186*w*, 1,053*w*, 907*w*, 819*w*, 777*w*, 758*w*, 729*w*, 679*w*; ^1^H-NMR (DMSO-*d*_6_), δ: 8.34–8.56 (m, 2H), 7.87–8.12 (m, 4H), 7.45–7.71 (m, 2H), 4.04 (qt, *J* = 8.8 Hz, *J* = 4.6 Hz, 1H), 1.81–2.03 (m, 2H), 1.29–1.77 (m, 10H); ^13^C-NMR (DMSO-*d*_6_), δ: 165.20, 134.05, 132.35, 132.17, 128.78, 127.69, 127.61, 127.41, 127.35, 126.63, 124.47, 50.59, 34.42, 27.89, 24.00; HR-MS: for C_18_H_22_NO [M+H]^+^ calculated 268.1696 *m/z*, found 268.1621 *m/z*.

*N-Cyclooctyl-2-naphthamide* (**27**). Yield 17%; Mp. 145–146 °C; IR (Zn/Se ATR, cm^−1^): 3,252*s*, 2,921*s*, 2,849*m*, 1,634*s*, 1,623*s*, 1,548*s*, 1,446*w*, 1,348*w*, 1,326*m*, 1,279*w*, 1,243*w*, 1,199*w*, 1,066*m*, 897*w*, 864*w*, 825*w*, 774*m*, 762*w*, 737*m*, 712*m*; ^1^H-NMR (DMSO-*d*_6_), δ: 8.30–8.55 (m, 2H), 7.87–8.10 (m, 4H), 7.46–7.69 (m, 2H), 4.09 (qt, *J* = 8.3 Hz, *J* = 4.2 Hz, 1H), 1.37–1.92 (m, 14H); ^13^C-NMR (DMSO-*d*_6_), δ: 165.18, 134.03, 132.37, 132.15, 128.76, 127.66, 127.59, 127.38, 127.32, 126.60, 124.47, 49.39, 31.72, 26.86, 25.14, 23.60; HR-MS: for C_19_H_24_NO [M+H]^+^ calculated 282.1852 *m/z*, found 282.1873 *m/z*.

*N-Phenyl-2-naphthamide* (**28**). Yield 96%; Mp. 175–176 °C (170 °C [50], Mp. 160 °C [54]); IR (Zn/Se ATR, cm^−1^): 3,360*m*, 3,256*w*, 3,053*w*, 2,989*w*, 1,640*s*, 1,595*s*, 1,519*s*, 1,489*s*, 1,431*s*, 1,313*m*, 1,245*w*, 1,131*w*, 1,076*w*, 956*w*, 912*m*, 870*w*, 824*w*, 777*w*, 749*s*, 731*m*, 689*m*; ^1^H-NMR (DMSO-*d*_6_) [55,56], δ: 10.48 (bs, 1H), 8.61 (s, 1H), 7.96–8.17 (m, 4H), 7.88 (d, *J* = 8.0 Hz, 2H), 7.56–7.71 (m, 2H), 7.39 (t, *J* = 7.5 Hz, 2H), 7.05–7.19 (m, 1H); ^13^C-NMR (DMSO-*d*_6_), δ: 165.66, 139.31, 134.30, 132.35, 132.12, 128.98, 128.69, 128.04, 128.03, 127.83, 127.71, 126.87, 124.53, 123.72, 120.42; HR-MS: for C_17_H_14_NO [M+H]^+^ calculated 248.1070 *m/z*, found 248.1095 *m/z*.

*N-Benzyl-2-naphthamide* (**29**). Yield 42%; Mp. 138–139 °C (Mp. 140–143 °C [57]); IR (Zn/Se ATR, cm^−1^): 3,287*s*, 3,054*w*, 3,027*w*, 2,920*w*, 1,635*s*, 1,622*s*, 1,600*w*, 1,543*s*, 1,504*m*, 1,495*m*, 1,449*w*, 1,414*m*, 1,359*w*, 1,316*m*, 1,300*m*, 1,263*m*, 1,206*w*, 1,146*w*, 1,119*w*, 1,047*w*, 996*w*, 910*m*, 869*m*, 835*m*, 777*m*, 737*w*, 719*m*, 693*m*; ^1^H-NMR (DMSO-*d*_6_), δ: 9.25 (t, *J* = 6.02 Hz, 1H), 8.53 (s, 1H), 7.91–8.11 (m, 4H), 7.53–7.70 (m, 2H), 7.29–7.46 (m, 4H), 7.21–7.28 (m, 1H), 4.56 (d, *J* = 6.02 Hz, 2H); ^13^C-NMR (DMSO-*d*_6_), δ: 166.29, 139.70, 134.17, 132.18, 131.72, 128.87, 128.32, 127.92, 127.63, 127.61, 127.55, 127.30, 126.77, 126.74, 124.22, 42.77; HR-MS: for C_18_H_16_NO [M+H]^+^ calculated 262.1226 *m/z*, found 262.1257 *m/z*.

*N-(2-Phenylethyl)-2-naphthamide* (**30**). Yield 26%; Mp. 133–134 °C (Mp. 133–134 °C [52]); IR (Zn/Se ATR, cm^−1^): 3,325*s*, 3,054*w*, 3,027*w*, 2,933*w*, 2,900*w*, 1,639*s*, 1,626*m*, 1,600*m*, 1,541*s*, 1,452*m*, 1,302*w*, 1,211*w*, 1,074*w*, 829*w*, 779*w*, 756*w*, 692*w*; ^1^H-NMR (DMSO-*d*_6_), δ: 8.78 (t, *J* = 5.5 Hz, 1H), 8.45 (s, 1H), 7.86–8.11 (m, 4H), 7.48–7.71 (m, 2H), 7.12–7.41 (m, 5H), 3.49–3.66 (m, 2H), 2.91 (t, *J* = 7.5 Hz, 2H); ^13^C-NMR (DMSO-*d*_6_), δ: 166.23, 139.58, 134.11, 132.17, 131.99, 128.84, 128.71, 128.38, 127.87, 127.63, 127.54, 127.37, 126.73, 126.13, 124.17, 41.06, 35.19; HR-MS: for C_19_H_18_NO [M+H]^+^ calculated 276.1383 *m/z*, found 276.1353 *m/z*.

*N-(3-Hydroxyphenyl)-2-naphthamide* (**31b**). Yield 69%; Mp. 194 °C; IR (Zn/Se ATR, cm^−1^): 3,349*s*, 1,712*s*, 1,663*s*, 1,603*s*, 1,530*s*, 1,432*s*, 1,277*s*, 1,220*s*, 1,192*s*, 1,144*s*, 1,081*s*, 970*s*, 913*s*, 861*s*, 820*s*, 772*s*, 760*s*, 679*s*; ^1^H-NMR (DMSO-*d*_6_), δ: 10.32 (s, 1H), 8.57 (s, 1H), 8.11–7.99 (m, 5H), 7.69–7.60 (m, 2H), 7.43 (t, *J* = 1.8 Hz, 1H), 7.25–7.10 (m, 2H), 6.54 (dt, *J* = 7.7 Hz, *J* = 2.2 Hz, 1H); ^13^C-NMR (DMSO-*d*_6_), δ: 165.36, 157.44, 140.13, 134.10, 132.34, 131.97, 129.04, 128.76, 127.75, 127.73, 127.58, 127.50, 126.62, 124.35, 111.09, 110.74, 107.51; HR-MS: for C_17_H_14_NO_2_ [M+H]^+^ calculated 264.1019 *m/z*, found 264.1012 *m/z*.

*N-(4-Hydroxyphenyl)-2-naphthamide* (**31c**). Yield 66%; Mp. 250–255 °C; IR (Zn/Se ATR, cm^−1^): 3,376*s*, 3,055*w*, 1,731*s*, 1,660*s*, 1,520*s*, 1,311*s*, 1,267*s*, 1,221*s*, 1,202*s*, 1,081*s*, 953*s*, 802*s*, 775*s*, 760*s*, 754*s*; ^1^H-NMR (DMSO-*d*_6_), δ: 10.58 (s, 1H), 8.61 (s, 1H), 8.14–7.99 (m, 5H), 7.70–7.61 (m, 2H), 7.40–7.34 (m, 2H), 6.79–6.74 (m, 2H); ^13^C-NMR (DMSO-*d*_6_), δ: 165.53, 153.65, 146.38, 134.21, 132.04, 131.36, 128.84, 127.93, 127.90, 127.66, 127.57, 124.32, 121.91, 121.31, 114.91; HR-MS: for C_17_H_14_NO_2_ [M+H]^+^ calculated 264.1019 *m/z*, found 264.1010 *m/z*.

*N-(2-Methoxyphenyl)-2-naphthamide* (**32a**). Yield 46%; Mp. 82 °C; IR (Zn/Se ATR, cm^−1^): 3,274*s*, 3,060*w*, 2,997*w*, 2,973*w*, 1,647*s*, 1,598*m*, 1,535*s*, 1,493*s*, 1,460*s*, 1,433*s*, 1,336*s*, 1,259*s*, 1,026*m*, 749*s*; ^1^H-NMR (DMSO-*d*_6_), δ: 9.62 (s, 1H), 8.62 (s, 1H), 8.10–8.08 (m, 1H), 8.04 (d, *J* = 1.0 Hz, 2H), 8.01 (dd, *J* = 8.2 Hz, *J* = 1.1 Hz, 1H), 7.85 (dd, *J* = 7.9 Hz, *J* = 1.3 Hz, 1H), 7.66–7.60 (m, 2H), 7.22–7.18 (m, 1H), 7.12–7.10 (m, 1H), 7.00 (td, *J* = 7.7 Hz, *J* = 1.2 Hz, 1H), 3.86 (s, 3H); ^13^C-NMR (DMSO-*d*_6_), δ: 165.08, 151.52, 134.30, 132.16, 131.84, 129.02, 128.12, 127.92, 127.84, 127.66, 126.89, 126.83, 125.76, 124.36, 124.24, 120.24, 111.41, 55.73; HR-MS: for C_18_H_16_NO_2_ [M+H]^+^ calculated 278.1176 *m/z*, found 278.1179 *m/z*.

*N-(3-Methoxyphenyl)-2-naphthamide* (**32b**). Yield 47%; Mp. 142 °C; IR (Zn/Se ATR, cm^−1^): 3,243*s*, 3,005*w*, 1,640*s*, 1,591*s*, 1,530*s*, 1,451*s*, 1,429*s*, 1,301*s*, 1,282*s*, 1,158*s*, 1,049*s*, 771*s*; ^1^H-NMR (DMSO-*d*_6_), δ: 10.43 (s, 1H), 8.59 (s, 1H), 8.11–8.07 (m, 1H), 8.05 (s, 1H), 8.04 (d, *J* = 1.5 Hz, 1H), 8.02–8.00 (m, 1H), 7.66–7.60 (m, 2H), 7.55 (t, *J* = 2.3 Hz, 1H), 7.45 (dd, *J* = 8.0 Hz, *J* = 1.0 Hz, 1H), 7.28 (t, *J* = 8.2 Hz, 1H), 6.71 (dd, *J* = 8.2 Hz, *J* = 1.8 Hz, 1H); 3.78 (s, 3H); ^13^C-NMR (DMSO-*d*_6_), δ: 165.44, 159.38, 140.30, 134.13, 132.16, 131.95, 129.20, 128.75, 127.81, 127.75, 127.61, 127.49, 126.64, 124.26, 112.51, 109.10, 106.06, 54.90; HR-MS: for C_18_H_16_NO_2_ [M+H]^+^ calculated 278.1176 *m/z*, found 278.1175 *m/z*.

*N-(4-Methoxyphenyl)-2-naphthamide* (**32c**). Yield 65%; Mp. 182 °C; IR (Zn/Se ATR, cm^−1^): 3,371*s*, 3,049*w*, 2,949*w*, 2,835*w*, 1,656*s*, 1,598*s*, 1,527*s*, 1,511*s*, 1,463*s*, 1,414*s*, 1,314*s*, 1,303*s*, 1,220*s*, 1,182*s*, 1,033*s*, 820*s*, 761*s*; ^1^H-NMR (DMSO-*d*_6_), δ: 10.34 (s, 1H), 8.58 (s, 1H), 8.08 (d, *J* = 8.8 Hz, 1H), 8.04 (s, 2H), 8.00 (d, *J* = 8.3 Hz, 1H), 7.75 (d, *J* = 8.3 Hz, 2H), 7.66–7.59 (m, 2H), 6.69 (d, *J* = 8.3 Hz, 2H), 3.76 (s, 3H); ^13^C-NMR (DMSO-*d*_6_), δ: 165.15, 155.57, 134.21, 132.39, 132.33, 132.12, 128.92, 127.97, 127.81, 127.72, 127.67, 126.81, 124.46, 121.99, 113.78, 55.18; HR-MS: for C_18_H_16_NO_2_ [M+H]^+^ calculated 278.1176 *m/z*, found 278.1173 *m/z*.

*N-(2-Methylphenyl)-2-naphthamide* (**33a**). Yield 33%; Mp. 146 °C; IR (Zn/Se ATR, cm^−1^): 3,239*s*, 3,057*w*, 1,639*s*, 1,584*s*, 1,531*s*, 1,454*s*, 1,315*s*, 1,134*s*, 916*s*, 777*s*, 749*s*, 731*s*, 692*m*; ^1^H-NMR (DMSO-*d*_6_), δ: 10.09 (s, 1H), 8.63 (s, 1H), 8.10–8.08 (m, 1H), 8.08–8.06 (m, 2H), 8.04–8.01 (m, 1H), 7.67–7.60 (m, 2H), 7.41 (d, *J* = 6.7 Hz, 1H), 7.30 (d, *J* = 7.3 Hz, 1H), 7.28 (td, *J* = 7.5 Hz, *J* = 1.5 Hz, 1H), 7.19 (td, *J* = 7.3 Hz, *J* = 1.3 Hz, 1H); 2.29 (s, 3H); ^13^C-NMR (DMSO-*d*_6_), δ: 166.05, 137.16, 134.95, 134.41, 132.82, 132.55, 131.02, 129.62, 128.69, 128.68, 128.42, 128.33, 127.49, 127.27, 126.70, 126.67, 125.12, 18.66; HR-MS: for C_18_H_16_NO [M+H]^+^ calculated 262.1226 *m/z*, found 262.1225 *m/z*.

*N-(3-Methylphenyl)-2-naphthamide* (**33b**). Yield 59%; Mp. 160 °C; IR (Zn/Se ATR, cm^−1^): 3,246*s*, 3,055*w*, 1,641*s*, 1,590*s*, 1,550*s*, 1,432*s*, 1,309*s*, 1,130*s*, 777*s*, 749*s*, 731*s*, 690*s*; ^1^H-NMR (DMSO-*d*_6_), δ: 10.37 (s, 1H), 8.59 (s, 1H), 8.10–8.07 (m, 1H), 8.04–8.02 (m, 2H), 8.02–8.00 (m, 1H), 7.69 (s, 1H), 7.66–7.60 (m, 3H), 7.26 (t, *J* = 7.8 Hz, 1H), 6.94 (d, *J* = 7.3 Hz, 1H), 2.33 (s, 3H); ^13^C-NMR (DMSO-*d*_6_), δ: 165.35, 139.02, 137.61, 134.12, 132.22, 131.97, 128.75, 128.28, 127.81, 127.72, 127.60, 127.49, 126.64, 124.29, 124.23, 120.84, 117.47, 21.03; HR-MS: for C_18_H_16_NO [M+H]^+^ calculated 262.1226 *m/z*, found 262.1224 *m/z*.

*N-(4-Methylphenyl)-2-naphthamide* (**33c**). Yield 67%; Mp. 195 °C (Mp. 191 °C [50]; IR (Zn/Se ATR, cm^−1^): 3,254*s*, 3,056*w*, 2,917*w*, 1,640*s*, 1,602*s*, 1,538*s*, 1,513*s*, 1,404*s*, 1,329*s*, 1,132*s*, 914*s*, 811*s*, 754*s*, 733*s*, 707*m*; ^1^H-NMR (DMSO-*d*_6_) [56], δ: 10.38 (s, 1H), 8.59 (s, 1H), 8.10–8.06 (m, 1H), 8.04 (s, 2H), 8.02–8.00 (m, 1H), 7.73 (d, *J* = 8.5 Hz, 2H), 7.66–7.60 (m, 2H), 7.18 (d, *J* = 8.3 Hz, 2H), 2.29 (s, 3H); ^13^C-NMR (DMSO-*d*_6_), δ: 165.22, 136.59, 134.10, 132.51, 132.27, 132.00, 128.85, 128.75, 127.79, 127.69, 127.57, 127.50, 126.64, 124.30, 120.34, 20.34; HR-MS: for C_18_H_16_NO [M+H]^+^ calculated 262.1226 *m/z*, found 262.1225 *m/z*.

*N-(2-Fluorophenyl)-2-naphthamide* (**34a**). Yield 59%; Mp. 122 °C; IR (Zn/Se ATR, cm^−1^): 3,316*s*, 3,054*w*, 3,023*w*, 1,648*s*, 1,541*s*, 1,489*s*, 1,456*s*, 1,321*s*, 1,257*s*, 1,192*m*, 829*m*, 750*s*; ^1^H-NMR (DMSO-*d*_6_), δ: 10.33 (s, 1H), 8.64 (s, 1H), 8.11–8.08 (m, 1H), 8.06 (d, *J* = 1.0 Hz, 2H), 8.03–8.01 (m, 1H), 7.70–7.60 (m, 3H), 7.36–7.23 (m, 3H); ^13^C-NMR (DMSO-*d*_6_), δ: 165.38, 155.64 (d, *J* = 245.8 Hz), 134.27, 131.98, 131.21, 128.82, 128.13, 127.88, 127.73, 127.50, 126.72 (d, *J* = 8.8 Hz), 126.68, 126.68 (d, *J* = 7.6 Hz), 125.75 (d, *J* = 11.4 Hz), 124.24, 124.13 (d, *J* = 3.8 Hz), 115.65 (d, *J* = 20.5 Hz); HR-MS: for C_17_H_13_NOF [M+H]^+^ calculated 266.0976 *m/z*, found 266.0974 *m/z*.

*N-(3-Fluorophenyl)-2-naphthamide* (**34b**). Yield 21%; Mp. 167 °C; IR (Zn/Se ATR, cm^−1^): 3,267*s*, 3,140*w*, 3,058*w*, 1,642*s*, 1,604*s*, 1,541*s*, 1,444*s*, 1,322*s*, 1,301*s*, 1,142*m*, 775*m*, 765*m*, 682*m*; ^1^H-NMR (DMSO-*d*_6_), δ: 10.64 (s, 1H), 8.59 (s, 1H), 8.11-8.09 (m, 1H), 8.08–8.01 (m, 3H), 7.83 (dt, *J* = 11.8 Hz, *J* = 2.5 Hz, 1H), 7.68–7.61 (m, 3H), 7.42 (dt, *J* = 8.2 Hz, *J* = 7.0 Hz, 1H), 6.95 (tdd, *J* = 8.5 Hz, *J* = 2.5 Hz, *J* = 0.75 Hz, 1H); ^13^C-NMR (DMSO-*d*_6_), δ: 165.71, 161.98 (d, *J* = 239.7 Hz), 140.89 (d, *J* = 11.4 Hz), 134.23, 131.93, 131.83, 130.06 (d, *J* = 9.9 Hz), 128.79, 127.93, 127.90, 127.75, 127.53, 126.73, 124.21, 115.90 (d, *J* = 3.1 Hz), 109.94 (d, *J* = 20.5 Hz), 106.90 (d, *J* = 25.8 Hz); HR-MS: for C_17_H_13_NOF [M+H]^+^ calculated 266.0976 *m/z*, found 266.0972 *m/z*.

*N-(4-Fluorophenyl)-2-naphthamide* (**34c**). Yield 70%; Mp. 191 °C; IR (Zn/Se ATR, cm^−1^): 3,380*s*, 3,063*w*, 1,654*s*, 1,527*s*, 1,506*s*, 1,405*s*, 1,313*m*, 1,212*s*, 1,197*s*, 826*s*, 814*s*, 764*s*; ^1^H-NMR (DMSO-*d*_6_) [56], δ: 10.51 (s, 1H), 8.58 (s, 1H), 8.10–8.08 (m, 1H), 8.07–8.00 (m, 3H), 7.88–7.84 (m, 2H), 7.66–7.60 (m, 2H), 7.25–7.20 (m, 2H); ^13^C-NMR (DMSO-*d*_6_), δ: 165.36, 158.22 (d, *J* = 238.9 Hz), 135.46 (d, *J* = 2.3 Hz), 134.17, 132.03, 131.98, 128.75, 127.84, 127.78, 127.64, 127.50, 126.67, 124.24, 122.13 (d, *J* = 8.0 Hz), 115.01 (d, *J* = 22.0 Hz); HR-MS: for C_17_H_13_NOF [M+H]^+^ calculated 266.0976 *m/z*, found 266.0975 *m/z*.

*N-(2-Chlorophenyl)-2-naphthamide* (**35a**) [58]. Yield 45%; Mp. 116 °C; IR (Zn/Se ATR, cm^−1^): 3,281*s*, 3,061*w*, 1,653*s*, 1,583*s*, 1,523*s*, 1,439*s*, 1,305*s*, 1,053*m*, 777*s*, 759*s*, 746*s*; ^1^H-NMR (DMSO-*d*_6_), δ: 10.26 (s, 1H), 8.65 (s, 1H), 8.11–8.09 (m, 1H), 8.09–8.05 (m, 2H), 8.04–8.00 (m, 1H), 7.68–7.61 (m, 3H), 7.59 (dd, *J* = 7.9 Hz, *J* = 1.3 Hz, 1H), 7.42 (td, *J* = 7.7 Hz, *J* = 1.5 Hz, 1H), 7.32 (td, *J* = 7.8 Hz, *J* = 1.5 Hz, 1H); ^13^C-NMR (DMSO-*d*_6_), δ: 165.48, 135.16, 134.42, 132.11, 131.30, 129.61, 129.55, 129.02, 128.50, 128.25, 128.15, 127.96, 127.70, 127.52, 127.51, 126.92, 124.34; HR-MS: for C_17_H_13_NOCl [M+H]^+^ calculated 282.0680 *m/z*, found 282.0679 *m/z*.

*N-(3-Chlorophenyl)-2-naphthamide* (**35b**). Yield 67%; Mp. 180 °C; IR (Zn/Se ATR, cm^−1^): 3,262*s*, 3,236*s*, 3,110*w*, 3,055*w*, 1,644*s*, 1,591*s*, 1,531*s*, 1,420*s*, 1,316*s*, 1,301*s*, 1,133*m*, 778*s*, 765*s*, 695*m*; ^1^H-NMR (DMSO-*d*_6_), δ: 10.61 (s, 1H), 8.59 (s, 1H), 8.11–8.09 (m, 1H), 8.08–8.01 (m, 4H), 7.78 (ddd, *J* = 8.3 Hz, *J* = 1.8 Hz, *J* = 0.8 Hz, 1H), 7.68–7.61 (m, 2H), 7.41 (t, *J* = 8.2 Hz, 1H), 7.18 (ddd, *J* = 8.0 Hz, *J* = 1.9 Hz, *J* = 0.8 Hz, 1H); ^13^C-NMR (DMSO-*d*_6_), δ: 165.69, 140.61, 134.24, 132.86, 131.93, 131.77, 130.14, 128.81, 127.95, 127.92, 127.78, 127.53, 126.75, 124.21, 123.21, 119.68, 118.55; HR-MS: for C_17_H_13_NOCl [M+H]^+^ calculated 282.0680 *m/z*, found 282.0681 *m/z*.

*N-(4-Chlorophenyl)-2-naphthamide* (**35c**). Yield 81%; Mp. 218 °C; IR (Zn/Se ATR, cm^−1^): 3,377*s*, 3,058*w*, 1,657*s*, 1,628*s*, 1,593*s*, 1,514*s*, 1,492*s*, 1,398*s*, 1,310*s*, 1,097*m*, 823*s*, 760*s*; ^1^H-NMR (DMSO-*d*_6_) [56], δ: 10.58 (s, 1H), 8.59 (s, 1H), 8.10–8.08 (m, 1H), 8.08–8.01 (m, 3H), 7.91–7.87 (m, 2H), 7.67–7.60 (m, 2H), 7.46–7.42 (m, 2H); ^13^C-NMR (DMSO-*d*_6_), δ: 165.54, 138.10, 134.21, 131.95, 131.92, 128.78, 128.38, 127.89, 127.88, 127.72, 127.52, 127.20, 126.72, 124.24, 121.78; HR-MS: for C_17_H_13_NOCl [M+H]^+^ calculated 282.0680 *m/z*, found 282.0679 *m/z*.

*N-(2-Bromophenyl)-2-naphthamide* (**36a**) [58]. Yield 70%; Mp. 123 °C; IR (Zn/Se ATR, cm^−1^): 3,277*s*, 3,060*w*, 1,652*s*, 1,629*m*, 1,578*m*, 1,523*s*, 1,433*s*, 1,304*s*, 1,028*m*, 777*m*, 759*m*, 745*s*; ^1^H-NMR (DMSO-*d*_6_), δ: 10.25 (s, 1H), 8.65 (s, 1H), 8.10-8.08 (m, 1H), 8.07 (s, 2H), 8.03-8.01 (m, 1H), 7.75 (dd, *J* = 7.8 Hz, *J* = 0.8 Hz, 1H), 7.68–7.61 (m, 3H), 7.46 (td, *J* = 7.5 Hz, *J* = 1.0 Hz, 1H), 7.25 (td, *J* = 7.7 Hz, *J* = 1.4 Hz, 1H); ^13^C-NMR (DMSO-*d*_6_), δ: 165.43, 136.70, 134.41, 132.73, 132.13, 131.41, 129.01, 128.93, 128.22, 128.15, 128.15, 127.95, 127.72, 126.94, 124.37, 124.34, 120.68; HR-MS: for C_17_H_13_NOBr [M+H]^+^ calculated 326.0175 *m/z*, found 326.0175 *m/z*.

*N-(3-Bromophenyl)-2-naphthamide* (**36b**). Yield 69%; Mp. 193 °C; IR (Zn/Se ATR, cm^−1^): 3,261*s*, 3,233*s*, 3,106*w*, 3,056*w*, 1,644*s*, 1,590*s*, 1,526*s*, 1,415*s*, 1,313*s*, 1,300*s*, 1,133*m*, 778*s*, 765*s*, 686*m*; ^1^H-NMR (DMSO-*d*_6_) [56], δ: 10.59 (s, 1H), 8.59 (s, 1H), 8.18 (t, *J* = 1.9 Hz, 1H), 8.11–8.08 (m, 1H), 8.08–8.00 (m, 3H), 7.83 (dt, *J* = 7.7 Hz, *J* = 1.6 Hz, 1H), 7.68–7.61 (m, 2H), 7.37–7.30 (m, 2H); ^13^C-NMR (DMSO‑*d*_6_), δ: 165.63, 140.74, 134.23, 131.92, 131.72, 130.43, 128.78, 127.93, 127.90, 127.75, 127.52, 126.73, 126.08, 124.20, 122.51, 121.25, 118.92; HR-MS: for C_17_H_13_NOBr [M+H]^+^ calculated 326.0175 *m/z*, found 326.0174 *m/z*.

*N-(4-Bromophenyl)-2-naphthamide* (**36c**). Yield 55%; Mp. 231 °C; IR (Zn/Se ATR, cm^−1^): 3,375*s*, 3,283*w*, 3,056*w*, 1,658*s*, 1,591*s*, 1,516*s*, 1,489*s*, 1,395*s*, 1,310*s*, 1,073*m*, 1,009*m*, 820*s*, 760*s*; ^1^H-NMR (DMSO-*d*_6_) [56], δ: 10.57 (s, 1H), 8.58 (s, 1H), 8.10–8.08 (m, 1H), 8.08–7.99 (m, 3H), 7.83 (d, *J* = 8.5 Hz, 2H), 7.67–7.61 (m, 2H) 7.57 (d, *J* = 8.5 Hz, 2H); ^13^C-NMR (DMSO-*d*_6_), δ: 165.54, 138.52, 134.21, 131.93, 131.90, 131.30, 128.79, 127.90, 127.72, 127.52, 126.72, 124.24, 122.16, 122.15, 115.21; HR-MS: for C_17_H_13_NOBr [M+H]^+^ calculated 326.0175 *m/z*, found 326.0173 *m/z*.

*N-(3-Trifluoromethylphenyl)-2-naphthamide* (**37b**). Yield 82%; Mp. 150 °C; IR (Zn/Se ATR, cm^−1^): 3,261*s*, 3,100*w*, 3,062*w*, 1,648*s*, 1,602*s*, 1,552*s*, 1,445*s*, 1,434*s*, 1,324*s*, 1,309*s*, 1,165*s*, 1,107*s*, 1,069*s*, 778*m*, 697*m*; ^1^H-NMR (DMSO-*d*_6_), δ: 10.75 (s, 1H), 8.63 (s, 1H), 8.32 (s, 1H), 8.15–8.01 (m, 5H), 7.69–7.60 (m, 3H), 7.47 (d, *J* = 7.8 Hz, 1H); ^13^C-NMR (DMSO-*d*_6_), δ: 165.85, 139.95, 134.29, 131.93, 131.66, 129.72, 129.31 (q, *J* = 32.1 Hz), 128.83, 128.02, 127.97, 127.82, 127.55, 126.78, 124.21, 124.06 (q, *J* = 273.1 Hz), 123.68, 119.79 (q, *J* = 3.8 Hz), 116.31 (q, *J* = 3.8 Hz); HR-MS: for C_18_H_13_NOF_3_ [M+H]^+^ calculated 316.0944 *m/z*, found 316.0942 *m/z*.

*N-(4-Trifluoromethylphenyl)-2-naphthamide* (**37c**). Yield 80%; Mp. 224 °C; IR (Zn/Se ATR, cm^−1^): 3,372*s*, 3,073*w*, 1,662*s*, 1,600*s*, 1,556*s*, 1,514*s*, 1,408*s*, 1,316*s*, 1,159*s*, 1,112*s*, 1,067*s*, 1,018*m*, 836*s*, 826*s*, 764*m*; ^1^H-NMR (DMSO-*d*_6_), δ: 10.78 (s, 1H), 8.62 (s, 1H), 8.12–8.01 (m, 6H), 7.76 (d, *J* = 8.8 Hz, 2H), 7.69–7.61 (m, 2H); ^13^C-NMR (DMSO-*d*_6_), δ: 165.97, 142.80, 134.32, 131.93, 131.71, 128.87, 128.13, 127.97, 127.87, 127.58, 126.81, 125.80 (q, *J* = 3.8 Hz), 124.29, 124.29 (q, *J* = 270.9 Hz), 123.57 (q, *J* = 32.1 Hz), 120.07; HR-MS: for C_18_H_13_NOF_3_ [M+H]^+^ calculated 316.0944 *m/z*, found 316.0942 *m/z*.

*N-(2-Nitrophenyl)-2-naphthamide* (**38a**). Yield 66%; Mp. 136 °C (Mp. 138–139 °C [59]); IR (Zn/Se ATR, cm^−1^): 3,383*s*, 3,124*w*, 3,054*w*, 1,678*s*, 1,582*s*, 1,493*s*, 1,448*s*, 1,427*s*, 1,335*s*, 1,284*s*, 1,273*s*, 1,195*s*, 759*s*, 738*s*; ^1^H-NMR (DMSO-*d*_6_), δ: 10.97 (s, 1H), 8.62 (s, 1H), 8.15–8.00 (m, 5H), 7.86–7.82 (m, 1H), 7.81–7.76 (m, 1H), 7.70–7.62 (m, 2H), 7.44 (t, *J* = 7.7 Hz, 1H); ^13^C-NMR (DMSO-*d*_6_), δ: 165.32, 142.68, 134.45, 133.94, 131.98, 131.63, 130.83, 128.92, 128.28, 128.20, 128.04, 127.60, 126.91, 125.80, 125.43, 124.88, 123.92; HR-MS: for C_17_H_13_N_2_O_3_ [M+H]^+^ calculated 293.0921 *m/z*, found 293.0919 *m/z*.

*N-(3-Nitrophenyl)-2-naphthamide* (**38b**). Yield 68%; Mp. 177 °C; IR (Zn/Se ATR, cm^−1^): 3,269*s*, 3,093*w*, 1,648*s*, 1,626*s*, 1,522*s*, 1,429*s*, 1,341*s*, 1,321*s*, 1,288*s*, 1,272*s*, 761*s*, 736*s*, 669*s*; ^1^H-NMR (DMSO-*d*_6_) [56], δ: 10.88 (s, 1H), 8.85 (t, *J* = 2.1 Hz, 1H), 8.63 (s, 1H), 8.26 (ddd, *J* = 8.2 Hz, *J* = 2.0 Hz, *J* = 0.9 Hz, 1H), 8.11–7.99 (m, 4H), 7.97 (ddd, *J* = 8.2 Hz, *J* = 2.0 Hz, *J* = 0.9 Hz, 1H), 7.69–7.61 (m, 3H); ^13^C-NMR (DMSO-*d*_6_), δ: 165.94, 147.88, 140.34, 134.35, 131.92, 131.42, 129.92, 128.85, 128.14, 128.02, 127.91, 127.58, 126.82, 126.06, 124.20, 117.96, 114.30; HR-MS: for C_17_H_13_N_2_O_3_ [M+H]^+^ calculated 293.0921 *m/z*, found 293.0917 *m/z*.

*N-(4-Nitrophenyl)-2-naphthamide* (**38c**). Yield 46%; Mp. 200 °C; IR (Zn/Se ATR, cm^−1^): 3,410*s*, 3,054*w*, 1,679*s*, 1,611*s*, 1,595*s*, 1,538*s*, 1,499*s*, 1,481*s*, 1,324*s*, 1,302*s*, 1,284*s*, 1,243*s*, 1,109*s*, 849*s*, 771*s*, 750*s*; ^1^H-NMR (DMSO-*d*_6_) [56], δ: 10.99 (s, 1H), 8.62 (s, 1H), 8.29 (dd, *J* = 9.3 Hz, *J* = 2.3 Hz, 2H), 8.13–8.01 (m, 6H), 7.69–7.61 (m, 2H); ^13^C-NMR (DMSO-*d*_6_), δ: 166.20, 145.45, 142.43, 134.41, 131.90, 131.43, 128.92, 128.37, 128.07, 128.02, 127.61, 126.88, 124.67, 124.27, 119.80; HR-MS: for C_17_H_13_N_2_O_3_ [M+H]^+^ calculated 293.0921 *m/z*, found 293.0918 *m/z*.

### 3.3. Study of Inhibition Photosynthetic Electron Transport (PET) in Spinach Chloroplasts

Chloroplasts were prepared from spinach (*Spinacia oleracea* L.) according to Masarovicova and Kralova [60]. The inhibition of photosynthetic electron transport (PET) in spinach chloroplasts was determined spectrophotometrically (Genesys 6, Thermo Scientific, USA), using an artificial electron acceptor 2,6-dichlorophenol-indophenol (DCIPP) according to Kralova *et al*. [61], and the rate of photosynthetic electron transport was monitored as a photoreduction of DCPIP. The measurements were carried out in phosphate buffer (0.02 mol/L, pH 7.2) containing sucrose (0.4 mol/L), MgCl_2_ (0.005 mol/L) and NaCl (0.015 mol/L). The chlorophyll content was 30 mg/L in these experiments and the samples were irradiated (~100 W/m^2^ with 10 cm distance) with a halogen lamp (250 W) using a 4 cm water filter to prevent warming of the samples (suspension temperature 22 °C). The studied compounds were dissolved in DMSO due to their limited water solubility. The applied DMSO concentration (up to 4%) did not affect the photochemical activity in spinach chloroplasts. The inhibitory efficiency of the studied compounds was expressed by IC_50_ values, *i.e.*, by molar concentration of the compounds causing 50% decrease in the oxygen evolution rate relative to the untreated control. The comparable IC_50_ value for a selective herbicide 3-(3,4-dichlorophenyl)-1,1-dimethylurea, DCMU (Diurone^®^) was about 1.9 μmol/L. The results are summarized in Table 1 and Table 2.

### 3.4. In Vitro Antimycobacterial Evaluation

Clinical isolates of *Mycobacterium tuberculosis* CUH071 (Cork University Hospital TB lab, with partial INH and PZA resistance), *M.* *avium* complex CIT19/06, *M. avium paratuberculosis* ATCC19698, and *M. kansasii* CIT11/06 were grown in Middlebrook broth (MB), supplemented with Oleic-Albumin-Dextrose-Catalase supplement (OADC, Becton Dickinson, UK). Identification of these isolates was performed using biochemical and molecular protocols. At log phase growth, culture (10 mL) was centrifuged at 15,000 rpm/20 min using a bench top centrifuge (Model CR 4-12, Jouan Inc., UK). Following removal of the supernatant, the pellet was washed in fresh Middlebrook 7H9GC broth and re-suspended in fresh supplemented MB (10 mL). The turbidity was adjusted to match McFarland standard No. 1 (3 × 10^8^ cfu) with MB broth. A further 1:20 dilution of the culture was then performed in MB broth.

The antimicrobial susceptibility of all four mycobacterial species was investigated in a 96-well plate format. In these experiments, sterile deionised water (300 µL) was added to all outer-perimeter wells of the plates to minimize evaporation of the medium in the test wells during incubation. Each evaluated compound (100 µL) was incubated with each of the mycobacterial species (100 µL). Dilutions of each compound were prepared in duplicate. For all synthesized compounds, final concentrations ranged from 1,000 µg/mL to 8 µg/mL. All compounds were prepared in DMSO and subsequent dilutions were made in supplemented MB. The plates were sealed with parafilm and incubated at 37 °C, for 5 days in the case of *M. kansasii* and *M. avium complex*, 7 days in the case of *M. tuberculosis* and 11 days in the case of *M. avium paratuberculosis*. Following incubation, a 10% addition of alamarBlue (AbD Serotec) was mixed into each well and readings at 570 nm and 600 nm were taken, initially for background subtraction and subsequently after 24 h re-incubation. The background subtraction is necessary for strongly coloured compounds, where the colour may interfere with the interpretation of any colour change. For non-interfering compounds, a blue colour in the well was interpreted as an absence of growth and a pink colour was scored as growth. The MIC was initially defined as the lowest concentration which prevented a visual colour change from blue to pink. Isoniazid (INH) and pyrazinamide (PZA) were used as the standards as it is a clinically used antimycobacterial drug. The MIC for mycobacteria was defined as a 90% or greater (IC_90_) reduction of growth in comparison with the control. The MIC/IC_90_ value is routinely and widely used in bacterial assays and is a standard detection limit according to the Clinical and Laboratory Standards Institute (CLSI, www.clsi.org). The results are summarized in Table 3.

### 3.5. *In Vitro* Cytotoxicity Assay

Human monocytic leukemia THP-1 cells were obtained from the European Collection of Cell Cultures (ECACC). These cells were routinely cultured in RPMI medium supplemented with 10% fetal bovine serum, 2% L-glutamine, 1% penicillin and streptomycin at 37 °C with 5% CO_2_. The tested compounds were dissolved in DMSO and added in four increasing concentrations to the cell suspension in the culture medium. Subsequently, the cells were incubated for 24 h at 37 °C with 5% CO_2_. Cell toxicity was determined using a WST-1 assay kit (Roche Diagnostics, Mannheim, Germany) according to the manufacturer’s instructions. For WST-1 assays, cells were seeded into 96-well plates (5 × 10^4^ cells·well^−1^ in 100 μL culture medium) in triplicate in serum-free RPMI 1640 medium, and measurements were taken 24 h after the treatment with tested compounds. The median lethal dose values, LD_50_, were deduced through the production of a dose-response curve. All data were evaluated using GraphPad Prism 5.00 software (GraphPad Software, San Diego, CA, USA, http://www.graphpad.com). The results are summarized in Table 3.

## 4. Conclusions

A series of thirty-five substituted quinoline-2-carboxamides and thirty-three substituted naphthalene-2-carboxamides were prepared and characterized. The prepared compounds were tested for their ability to inhibit photosynthetic electron transport (PET) in spinach chloroplasts (*Spinacia oleracea* L.) and for their antituberculosis/antimycobacterial activity. Two compounds, *N-*benzyl-2-naphthamide (**29**) and *N-*(2-hydroxyphenyl)quinoline-2-carboxamide (**12a**) showed relatively high PET inhibition. *N*-(2-Phenylethyl)quinoline-2-carboxamide (**11**), *N*-cycloheptylquinoline-2-carboxamide (**7**) and *N-*cyclohexylquinoline-2-carboxamide (**6**) expressed high activity against *Mycobacterium tuberculosis*. 1-(2-Naphthoyl)pyrrolidine (**22**) and 2-(pyrrolidin-1-ylcarbonyl)quinoline (**3**) showed high activity against *M. kansasii* and *M. avium paratuberculosis*. All five compounds exhibited activity comparable with or higher than the standards isoniazid or pyrazinamide. Lipophilicity was fundamental for the biological activities of all compounds in both biological assays. It can be stated that the dependence of PET-inhibiting activity on the lipophilicity decreases with increasing lipophilicity, while antimycobacterial activity increases with lipophilicity increase. Highly effective compounds against *M. tuberculosis* were detected, namely quinaldinamides, while naphtamides were more active against the other mycobacterial species. Substituted *N*-quinaldinanilides and/or *N*-naphtanilides seem to be less effective than other discussed *N*-nonaromatic amide derivatives. The most effective antimycobacterial compounds **11**, **7**, **3** and **22** were tested for their *in vitro* cytotoxicity against THP-1 cells. According to the calculated selectivity index of compounds **11**, **3** and **22** it can be concluded that the discussed amides can be considered as promising agents for subsequent design of novel antitubercular/ antimycobacterial agents.
